# Comprehensive analysis of cuproptosis-related prognostic gene signature and tumor immune microenvironment in HCC

**DOI:** 10.3389/fgene.2023.1094793

**Published:** 2023-02-20

**Authors:** Haotian Qin, Weibei Sheng, Geng Zhang, Qi Yang, Sen Yao, Yaohang Yue, Peng Zhang, Yuanchao Zhu, Qichang Wang, Yixiao Chen, Hui Zeng, Jian Weng, Fei Yu, Jun Yang

**Affiliations:** ^1^ National and Local Joint Engineering Research Center of Orthopaedic Biomaterials, Peking University Shenzhen Hospital, Shenzhen, China; ^2^ Department of Bone and Joint Surgery, Peking University Shenzhen Hospital, Shenzhen, China; ^3^ Zunyi Medical University, Zunyi, China; ^4^ Department of Medical Ultrasound, Peking University Shenzhen Hospital, Shenzhen, China; ^5^ Department of Radiology, Peking University Shenzhen Hospital, Shenzhen, China

**Keywords:** cuproptosis-related genes, hepatocellular carcinoma, prognosis, bioinformatics analysis, immune microenvironment

## Abstract

**Background:** Copper is an indispensable mineral element involved in many physiological metabolic processes. Cuproptosis is associated with a variety of cancer such as hepatocellular carcinoma (HCC). The objective of this study was to examine the relationships between the expression of cuproptosis-related genes (CRGs) and tumor characteristics, including prognosis and microenvironment of HCC.

**Methods:** The differentially expressed genes (DEGs) between high and low CRGs expression groups in HCC samples were identified, and further were analyzed for functional enrichment analysis. Then, CRGs signature of HCC was constructed and analyzed utilizing LASSO and univariate and multivariate Cox regression analysis. Prognostic values of CRGs signature were evaluated by Kaplan-Meier analysis, independent prognostic analysis and nomograph. The expression of prognostic CRGs was verified by Real-time quantitative PCR (RT-qPCR) in HCC cell lines. In addition, the relationships between prognostic CRGs expression and the immune infiltration, tumor microenvironment, antitumor drugs response and m6A modifications were further explored using a series of algorithms in HCC. Finally, ceRNA regulatory network based on prognostic CRGs was constructed.

**Results:** The DEGs between high and low CRG expression groups in HCC were mainly enriched in focal adhesion and extracellular matrix organization. Besides, we constructed a prognostic model that consists of CDKN2A, DLAT, DLST, GLS, and PDHA1 CRGs for predicting the survival likelihood of HCC patients. And the elevated expression of these five prognostic CRGs was substantially in HCC cell lines and associated with poor prognosis. Moreover, immune score and m6A gene expression were higher in the high CRG expression group of HCC patients. Furthermore, prognostic CRGs have higher mutation rates in HCC, and are significantly correlated with immune cell infiltration, tumor mutational burden, microsatellite instability, and anti-tumor drug sensitivity. Then, eight lncRNA-miRNA-mRNA regulatory axes that affected the progression of HCC were predicted.

**Conclusion:** This study demonstrated that the CRGs signature could effectively evaluate prognosis, tumor immune microenvironment, immunotherapy response and predict lncRNA-miRNA-mRNA regulatory axes in HCC. These findings extend our knowledge of cuproptosis in HCC and may inform novel therapeutic strategies for HCC.

## Introduction

Hepatocellular carcinoma (HCC) accounts for approximately 90% of liver cancers and is a common cause of death in cancer patients (Villanueva, 2019). The World Health Organization estimates that more than 1 million patients will die of liver cancer by 2030, according to annual projections (Villanueva, 2019). Liver cancer has a 5-year survival rate of 18% and is the second most deadly cancer after pancreatic cancer (Jemal et al., 2017). Effective treatments for advanced liver cancer are currently unavailable, and most patients can only use a palliative therapy such as chemoembolization. Therefore, it is necessary for us the development of new methods to effectively treat liver cancer is of utmost importance (Liver and Cancer, 2012; Llovet et al., 2016).

Previous studies showed that a variety of therapeutic methods involving programmed cell death such as pyroptosis and ferroptosis play an important role in inhibiting the occurrence and development of tumors (Fang et al., 2020; Li and Li, 2020; Tan et al., 2020). Among them, ferroptosis plays an important role in inhibiting tumor growth, and the enrichment of intracellular iron ions enhances the therapeutic effect of anti-cancer drugs (Li and Li, 2020). Pyroptosis also has effects on tumor cell proliferation, invasion, and metastasis, thereby affecting cancer prognosis (Fang et al., 2020; Tan et al., 2020). Copper is an indispensable trace element involved in various biological processes. The content of copper in the human body under normal circumstances is maintained at a steady state, but the content is modified under pathological conditions. For example, the content of copper in tumor tissue and serum in various cancers is significantly increased compared with the content in normal tissues (Yaman et al., 2007; Baharvand et al., 2014; Dragutinović et al., 2014; Kaba et al., 2015). The dysregulation of copper storage induces oxidative stress and cytotoxicity, while the effective regulation of copper content can affect cancer progression (Davis et al., 2020; Golonka and Vijay-Kumar, 2021). Based on this principle, a variety of copper chelators and copper ionophores have been developed for the replacement of therapies against tumors (Brady et al., 2014; Safi et al., 2014; Jemal et al., 2017; Roth and Decaens, 2017).

Recently, Tsvetkov et al. (2022) discovered a new copper-dependent mode of controlled cell death that differs from known cell death forms: copper can bind directly to fatty acylation components of the tricarboxylic acid cycle (TCA), leading to proteotoxic stress and ultimately cell death. This process is called cuproptosis, and cuproptosis-related genes (CRGs) were also identified. The Cyclin-Dependent Kinase Inhibitor 2A/Multiple Tumor Suppressor 1 (CDKN2A) gene, also known as P16 gene, encodes multiple tumor suppressor 1 and belongs to the INK4 family (Serrano, 1997). Dihydrolipoamide S-acetyltransferase (DLAT) and dihydrolipoamide S-succinyltransferase (DLST) are components of the pyruvate dehydrogenase (PDH) complex. The oligomerization of DLAT is due to the integration of copper and fatty acylated proteins in the TCA (Tsvetkov et al., 2022). Glutaminase (GLS) is an enzyme that converts glutamine to glutamate. Glutamine generates ATP by entering the TCA cycle through the mitochondrial oxidative phosphorylation or by recycling reducing equivalents through lactate secretion (glutaminolysis), thereby contributing to the production of energy and building blocks in cancer cells (Le et al., 2012; Fendt et al., 2013; Zhang et al., 2019). Pyruvate dehydrogenase E1 subunit alpha 1 (PDHA1) is a key component of PDH, which converts pyruvate to acetyl-CoA (Ozden et al., 2014). Deficiency of PDHA1 leads to mitochondrial dysfunction and promotes glycolysis (Zhong et al., 2015). PDHA1 has an important biological significance in a variety of human tumors (Song et al., 2019). Seven genes (FDX1, LIAS, LIPT1, DLD, DLAT, PDHA1, and PDHB) conferred resistance tocuproptosis, while three genes (MTF1, GLS, and CDKN2A) sensitized the cells to cuproptosis (Tsvetkov et al., 2022). Copper importers (SLC31A1) and copper exporters (ATP7A andATP7B) are important factors in keeping the intracellular copper concentration (Lutsenko, 2010). Mutations in ATP7A and ATP7B genes were found to cause Menke’s disease and Wilson’s disease (Nevitt et al., 2012). NLRP3 may influence tumor immunity mainly by mediating tumor-infiltrating lymphocytes and macrophages (Ju et al., 2021). NFE2L2 pathway is an important modulator of cell homeostasis, associated with enhanced tumor growth and aggressiveness (Hellyer et al., 2021). However, only few studies on CRGs in HCC are currently available. The study of the role of CRGs in HCC might be potentially beneficial for the development of new targets in the treatment of HCC.

This study comprehensively investigated the molecular alterations and clinical relevance of CRGs in HCC. A total of 371 HCC patients were selected from The Cancer Genome Atlas (TCGA) database, classified into 2 cuproptosis-related subtypes by consensus clustering based on 19 CRGs, the signaling pathways of differential gene enrichment in the two subtypes were analyzed, and the differences in immune cell infiltration, important immune checkpoints, and m6A methylation-related genes between the two subtypes were measured. The CRGs associated with the prognosis of HCC patients were also analyzed by logrank test and univariate regression analysis. Gene and protein expression of prognostic CRGs in HCC cells or tissues were also assessed, as well as their association with anti-tumor drug sensitivity, m6A methylation-related genes, and tumor microenvironment (TME). Prognostic models were developed to predict the overall survival (OS) and progression-free survival (PFS) in patients with HCC. A competing endogenous RNA (ceRNA) regulatory network was constructed to screen the lnRNA-miRNA-mRNA network that affected the prognosis of HCC patients. Our analysis highlighted the importance of CRGs in HCC development and laid the foundation for the therapeutic application of cuproptosis regulators in HCC.

## Materials and methods

### Data sources and preprocessing

CRGs and clinical information of patients with HCC were obtained from the TCGA database (https://portal.gdc.cancer. Gov/) (Tomczak et al., 2015). A total of 371 HCC tissues and 50 paracancerous tissues were collected and used in this study. The data used in this study were standardized data per million transcripts, and the data distribution was close to normal distribution. R software (v4.0.3) was used to extract gene expression data, and a data matrix was constructed for further analysis.

### Identification of molecular subgroups

Nineteen CRGs were first extracted from the TCGA expression matrix. According to the consistent clustering of these 19 genes, the R software package Consensus-ClusterPlus (v1.54.0) was used for the analysis of consistency, and the maximum number of clusters was 6 (Wilkerson and Hayes, 2010). The TCGA-LIHCREAD cases were then divided into 2 clusters based on the expression profiles of CRGs. This process was repeated 500 times to ensure the stability and reproducibility of the classification.

### Identification and functional enrichment analysis of DEGs

DEGs between C1 and C2 subgroups were obtained using Limma package (version 3.40.2) in R software (Ritchie et al., 2015). Adjusted *p* values were analyzed in the TCGA database to correct for false positive results. “Adjusted *p* < 0.05 and log2 (fold change) > 1 or log2 (fold change) < −1″ was defined as the criteria for screening the differential expression of mRNA. GeneMANIA (http://www.genemania.org), which is a software that elucidates the relationship between genes and datasets by building gene interaction networks (Warde-Farley et al., 2010), was used to visualize the gene network of CRGs through physical interaction, co-expression, prediction, co-localization, and genetic interaction, and to evaluate their functions.

The GO function and the enrichment of KEGG pathways were analyzed by the “clusterProfiler” package in R software (Yu et al., 2012). Potential biological pathways were also identified by GSEA (http://software.broadinstitute.org/gsea/index.jsp) (Powers et al., 2018). According to the data of TCGA, the DEGs were divided into upregulated and downregulated groups. Each analysis performed 10,000 gene combination permutations to identify signaling pathways with significant changes; the genes were considered as enriched for meaningful signaling pathways when *p*.adjust <0.05 and FDR (false discovery rate) < 0.25. Statistical analysis and graphing were performed using the R package clusterProfiler (3.18.0).

### Immune infiltration, and immune checkpoint-related genes expression in two subgroups

The R software package immunedeconv was used for immune score assessment to compare the degree of immune cell infiltration in C1 (N = 252) and C2 (N = 119) subgroups by Wilcoxon test, by integrating six state-of-the-art algorithms, including TIMER (Li et al., 2016), xCell (Aran et al., 2017), MCP-counter (Becht et al., 2016), CIBERSORT (Newman et al., 2015), EPIC (Racle et al., 2017) and quantTIseq (Finotello et al., 2019; Sturm et al., 2020). The expression of some immune checkpoint-related genes was also analyzed. The results were visualized by the R (v4.0.3) packages “ggplot2″ and “pheatmap".

### Expression of CRGs and survival analysis

The expression of CRGs in 371 HCC tissues and 50 paired adjacent tissues was analyzed using the TCGA database. In addition, univariate Cox regression analysis was used to investigate the effect of CRGs on the prognosis of HCC. Kaplan-Meier curves, *p* values, and hazard ratios (HR) with 95% confidence intervals (CIs) were obtained by logrank test and univariate Cox regression. Five CRGs (*CDKN2A, DLAT, DLST, GLS,* and *PDHA1*) with higher hazard ratios were screened from the Cox regression analysis plot. The relationship between the prognostic CRGs and the OS rate in HCC patients was also analyzed, and the AUC (Area Under Curve) under the ROC curve was calculated.

### Cell lines and culture conditions

The following HCC cell lines and hepatocyte were used in this study: Hep3B, Huh7, HepG2, and L02 (Chinese Academy of Medical Sciences, Beijing, China). All cell lines were maintained in Dulbecco’s modified Eagle’s medium (DMEM; Gibco, Grand Island, NY, United States) supplemented with 10% fetal bovine serum (Gibco, Grand Island, NY, United States of), 100 U/ml penicillin, and 100 U/ml streptomycin (Invitrogen, Carlsbad, CA, United States) and incubated at 37°C in a humidified atmosphere with 5% CO_2_.

### Quantitative RT-PCR

First, TRIzol reagent (Invitrogen, CA, United States) was used to extract total RNA from the samples. Then, the total RNA concentrations were measured using a NanoDrop 2000c (Thermo Fisher Scientific, Inc.). Next, cDNA was synthesized by reverse transcription using a miScript II RT kit (Qiagen, Germany). Finally, a miScript SYBR Green PCR kit (Qiagen, Germany) was used to detect the expression of target genes on a Lightcycler 480 Real-Time PCR System (Roche Diagnostics GmbH, Mannheim, Germany). The 20-μl reaction mixture included 10 μl 2X QuantiTect SYBR Green PCR Master Mix, 0.8 μl 10X miScript universal Primer, 2 μl cDNA template, 0.8 μl specific miRNA primer, and 7.4 μl RNase-free water. Additionally, the thermocycling steps were as follows: 95°C for 15 min, followed by 40 cycles of 94°C for 15 s, 55°C for 30 s, and 72°C for 30 s. The relative standard curve method (2^−△△CT^) was employed to determine the relative mRNA expression, with the glyceraldehyde 3-phosphate dehydrogenase (GAPDH) gene as the reference. Supplementary Table S1 lists the polymerase chain reaction primers used in this study.

### Development of the CRG prognostic model

A prognostic model was constructed using LASSO-Cox regression analysis based on the above CRGs associated with the prognosis of HCC patients. According to the results of the multivariate Cox regression analysis, the prognostic CRG risk score was calculated as follows: Risk score = ∑i Cofficient(mRNAi) × Expression (mRNAi). Then, TCGA-LIHC patients were divided into low-risk and high-risk subgroups according to the average risk score, Kaplan-Meier analysis was used to compare the OS rates of the two subgroups, and timeROC analysis was performed to predict the accuracy of the model. Each variable (including the *p*-value, and HR with 95% CI) was displayed by univariate and multivariate cox regression analysis and using forest plots by the “forestplot” package. The “rms” package was used to build a Nomogram model for predicting 1, 3, and 5-year OS and PFS based on the results of multivariate cox proportional hazards analysis.

### Tumor staging analysis of HCC

GEPIA is a newly developed interactive website for analyzing RNA sequencing expression data from 9736 tumors and 8587 normal samples from TCGA and Genotype-Tissue Expression databases, such as differential expression analysis of tumor/normal tissues, tumor type or pathological stage, patients’ survival analysis, similar gene detection analysis, and dimensionality reduction analysis (Tang et al., 2017). The expression data of prognostic CRGs in different stages of HCC were obtained using the “Stage Plot” module in the GEPIA2 database (http://gepia2.cancer-pku.cn/#index). The UALCAN database (http://ualcan.path.uab.edu/) is an interactive portal containing TCGA RNA transcriptome data and clinical data from 31 cancer types. TISIDB (http://cis.hku.hk/TISIDB) is a portal for assessing tumors and the immune system that integrates multiple heterogeneous data types including genomics, transcriptomics, and clinical data from TCGA of 30 cancer types (Ru et al., 2019). The UALCAN and TISIDB databases were used to confirm the association between prognostic CRGs and the clinical stage of HCC patients. *p* < 0.05 was considered statistically significant.

### Immunohistochemistry of prognostic CRGs in HCC

Immunohistochemical images of CRGs were collected from the Human Protein Atlas (https://www.proteinatlas.org) database to assess the differences in CRG expression between HCC and adjacent tissues (Sun et al., 2021).

### Mutation analysis of CRGs

cBioPortal (http://www.cbioportal.org/) provides visualization tools for analyzing cancer genetic data. The genomic maps of CRGs were analyzed using cBioPortal based on the TCGA database to understand the mutation frequency in HCC (Gao et al., 2013). In this study, 372 LIHC samples was selected to explore the impact of prognostic CRGs on survival of HCC patients. In addition, the effects of CRG mutations on cancer signaling pathway expression, TMB, MSIsensor scores, and hypoxia-related scores (Ragnum Hypoxia Score, Buffa Hypoxia Score, and Winter Hypoxia Score) were examined. *p* < 0.05 was considered statistically significant.

### Effects of prognostic CRGs on immune cell infiltration and immune checkpoint expression

The effects of prognostic CRGs on the abundance of infiltrating immune cells in tumors were analyzed by the TIMER (https://cistrome.shinyapps.io/timer/) database (Li et al., 2017). The detected immune cells included tumor purity, B cells, CD4^+^ T cells, CD8^+^ T cells, neutrophils, macrophages, and dendritic cells. In addition, the infiltration of immune cell types was quantified by the R package “GSVA” single sample gene set enrichment analysis (ssGSEA) (Hanzelmann et al., 2013). Next, the Spearman method was used to explore the correlation of prognostic CRGs with the level of immune cell infiltration. Finally, the relationship between prognostic CRGs and checkpoints (CD274, CTLA4, and PDCD1) was examined.

### TMB, microsatellite instability and drug sensitivity

The Spearman’s method was used to analyze the correlation between prognostic CRGs and MSI in HCC. The chemotherapeutic response was predicted for each sample using the GDSC database (https://www.cancerrxgene.org/) (Yang et al., 2013). The R package pRRophetic was used to predict the half maximum inhibitory concentration (50% inhibition of the concentration, IC50) of the chemotherapeutic drugs, in which the IC50 of the sample was estimated by ridge regression. All parameters were set by default, using the batch effect of combat and the organization types of all, and summarizing the replicate gene expression as an average. Drug sensitivity and gene expression profiling data from cancer cell lines in the GDSC database were integrated in this study.

### m6A-related gene expression analysis

The correlation between 19 CRGs and m6A-related genes expression and prognostic in 371 HCC samples was analyzed by R (4.0.3) packages “ggplot2.” The difference in m6A-related gene expression between C1 group (N = 252) and C2 group (N = 119) was investigated. The m6A-related genes analyzed included METTL3, YTHDC1, YTHDC2, METTL14, RBM15, RBM15B, IGF2BP1, IGF2BP2, IGF2BP3, VIRMA, WTAP, YTHDF1, YTHDF2, YTHDF3, ZC3H13, HNRNPA2B1, HNRNPC, RBMX, FTO, and ALKBH5.

### Single cell analysis

The effect of prognostic CRGs on the expression of single cell subsets in the TME was investigated using TISCH (http://tisch.comp-genomics.org/) (Sun et al., 2021). TISCH is a scRNA-seq database focused on the TME, providing detailed cell-type annotation at the single-cell level, which is beneficial for exploring the TME in different cancer types. In this dataset, three main cell types are present, such as immune cells, stromal cells, and malignant cells. In this study, the t-distributed stochastic neighborhood embedding (t-SNE) map of LIHC_GSE125449_aPDL1aCTLA4 and the heatmap of LIHC_GSE125449_aPDL1aCTLA4 were displayed through the TISCH database to demonstrate the effect of CRGs on the TME of HCC.

### Competing endogenous RNA network construction

Potential miRNA targets of prognostic CRGs were predicted using the ENCORI (http://starbase.sysu.edu.cn/) database (Li et al., 2014), RNAInter (http://www.rnainter.org/) (Lin et al., 2020) and RNA22 (https://cm.jefferson.edu/rna22/interactive) database (Loher and Rigoutsos, 2012), and the prognostic value of these potential miRNA targets in HCC was confirmed using ENCORI, Kaplan-Meier Plotter and TCGA-LIHC cohort. Then, the lncRNAs potentially binding to prognostic miRNAs were predicted by the miRNet (http://www.mirnet.ca/) database (Chang et al., 2020). Subsequently, a miRNA-lncRNA regulatory network was established by Cytoscape (version 3.7.1; http://www.cytoscape.org/) software (Shannon et al., 2003). In addition, the prognostic value of these potential lncRNA targets in HCC were further verified. Finally, a lncRNA-miRNA-mRNA regulatory network was established.

## Results

### Identification and analysis of cuproptosis-related gene clusters in HCC

**FIGURE 1:**
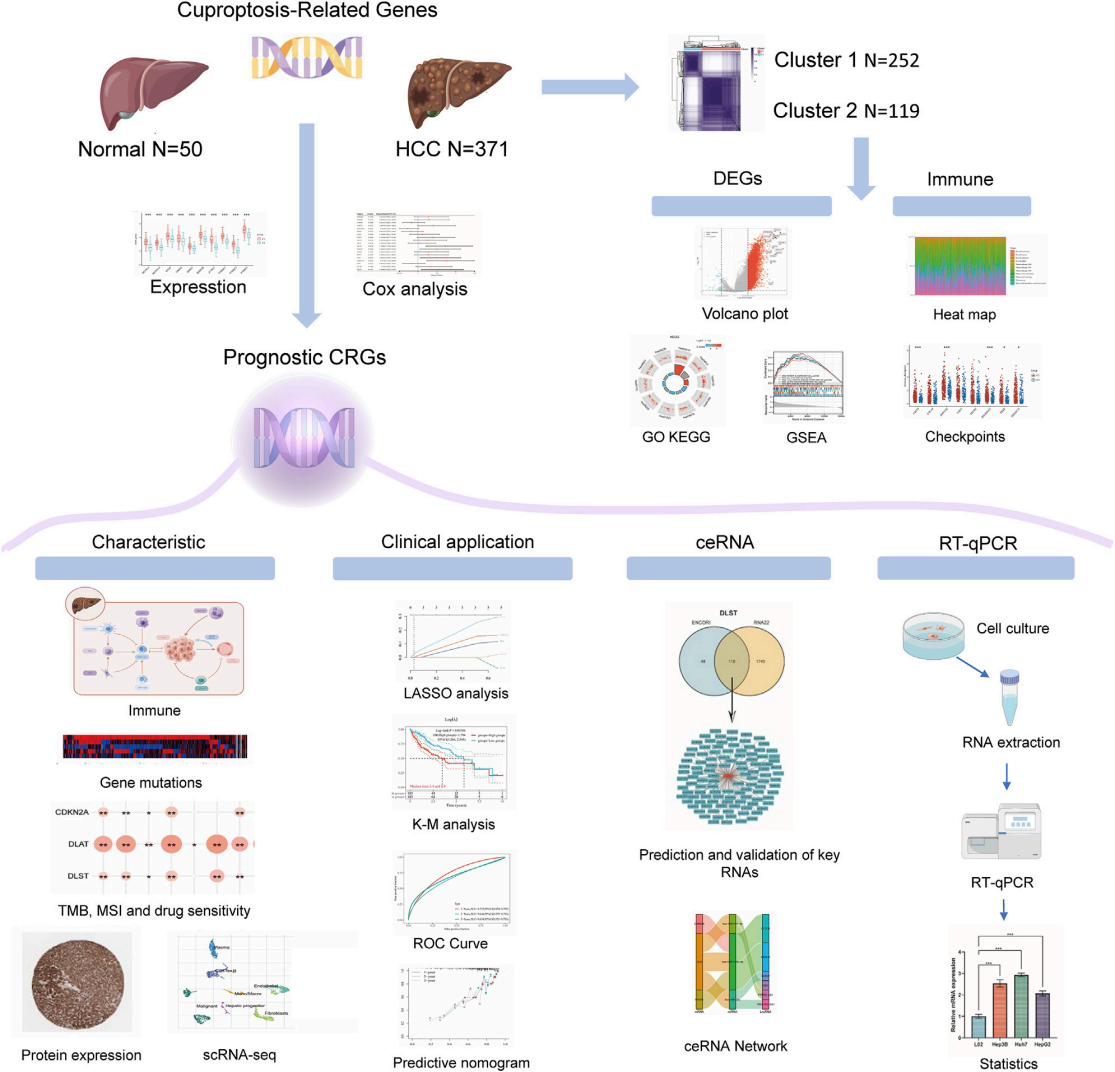
The flowchart of the present study.

**FIGURE 2:**
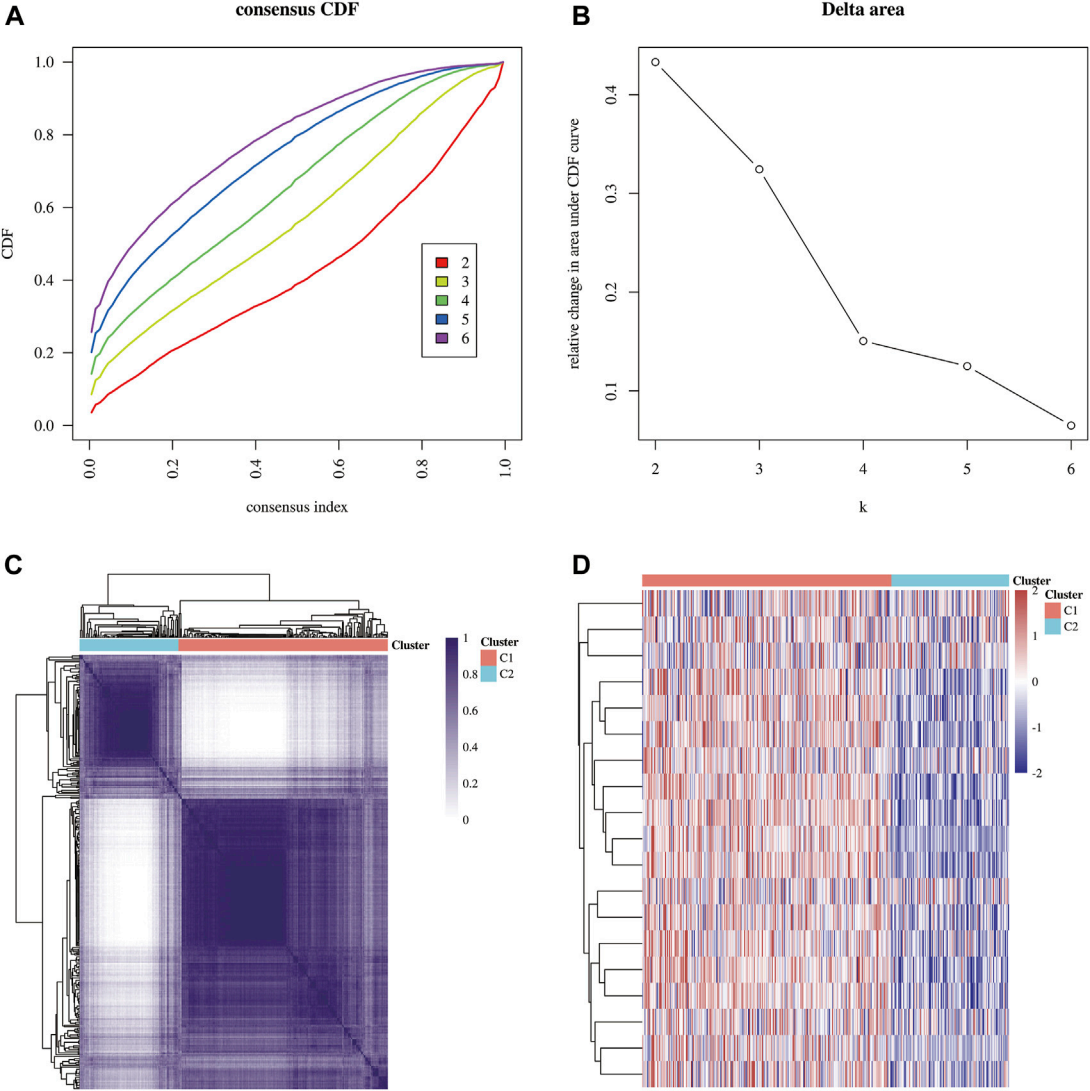
Identification of common clusters based on the expression of CRGs. **(A)** Cumulative distribution function (CDF) (k = 2–6). **(B)** Relative change of the area under the CDF curve (k = 2–6). **(C)** Consensus clustering matrix (k = 2). **(D)** Heat map of cuproptosis-related gene expression in different subgroups; red for high expression and blue for low expression.

The flowchart of the study is illustrated in Figure 1. A total of 371 HCC carcinoma samples were clustered in the TCGA database using consensus clustering T = to identify potential CRG clusters. All tumor samples were divided into k (k = 2–6) distinct clusters based on the expression of 19 CRGs in HCC (Figures 2A,B). Then, the number of clusters was selected as 2 according to the cluster analysis results, indicating that the patients with HCC were accurately divided into two clusters (Cluster 1 had 252 patients and Cluster 2 had 119 patients) (Figure 2C). Figure 2D shows the heat map of cuproptosis-related gene expression in different subgroups.

### Differentially expressed genes and functional enrichment analysis

**FIGURE 3:**
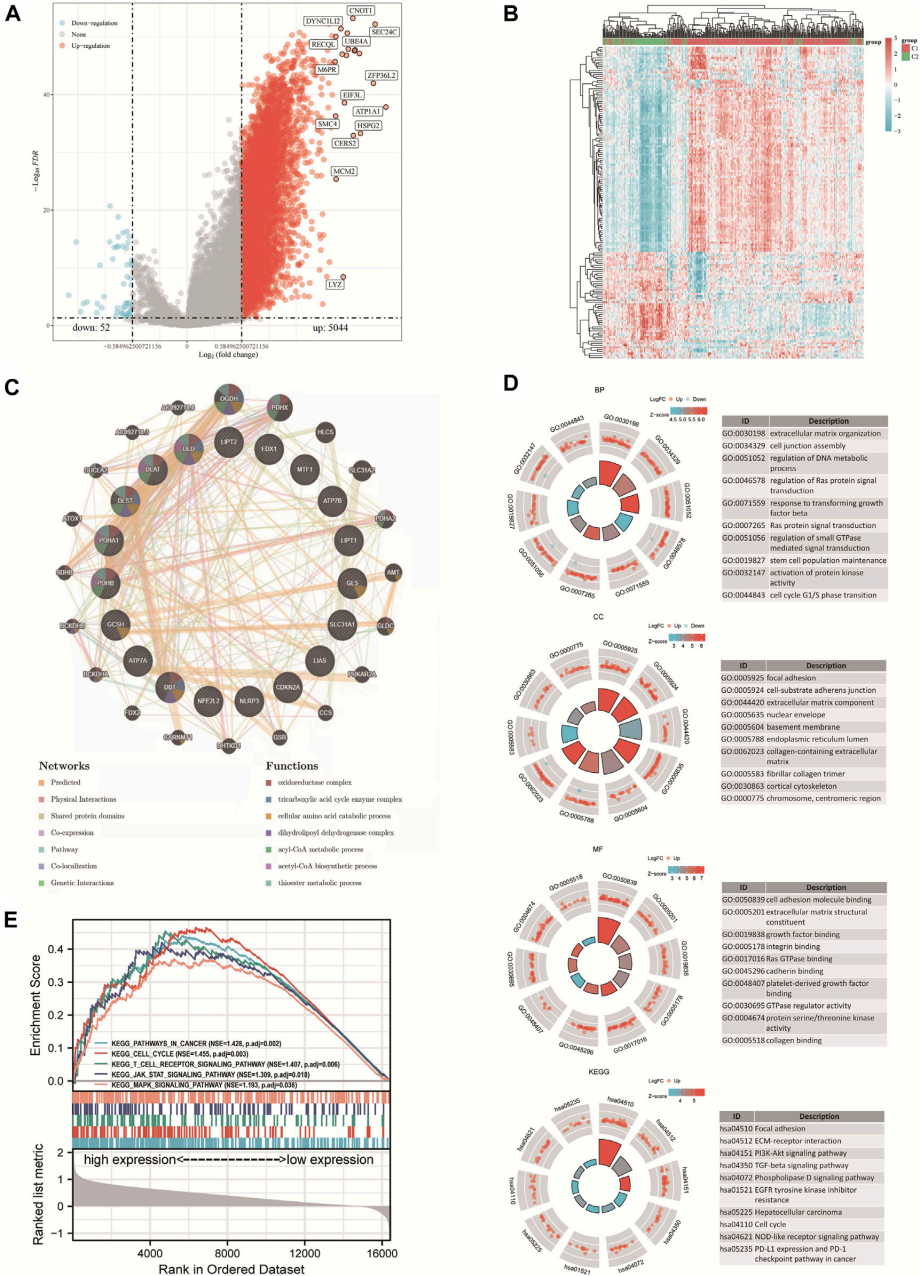
DEGs and functional enrichment analysis. **(A)** DEGs’ volcano plot between C1 and C2 subtypes. **(B)** DEGs’ heat map between C1 and C2 subtypes. **(C)** Gene Interaction Network. **(D)** Enriched item in gene ontology (GO) analysis and Kyoto Encyclopedia of Genes and Genomes analysis (KEGG). **(E)** Enrichment plots from GSEA. BP, biological process; CC, cellular composition; MF, molecular function.

The study included 421 liver HCC samples consisting of 371 tumor samples and 50 adjacent normal samples. The DEGs identified between the C1 and C2 subtypes contained 5044 upregulated genes and 52 downregulated genes. Then, a volcano map (Figure 3A) and a heat map (Figure 3B) were constructed based on these DEGs. The results of GeneMANIA revealed that the functions of the significant genes co-expressed in this network (*DLD, GLS, NFE2L2, DLST, LIAS, ATP7B, PDHA1, SLC31A1, FDX1, DBT, GCSH, LIPT1, CDKN2A, DLAT, NLRP3, ATP7A, PDHB, LIPT2,* and *MTF1*) were related to several processes, including oxidoreductase complex, tricarboxylic acid cycle enzyme complex, cellular amino acid catabolic process, dihydrolipoyl dehydrogenase complex, acyl-CoA metabolic process, acetyl-CoA biosynthetic process and thioester metabolic process (Figure 3C). The identified up and downregulated DEGs were then further subjected to GO and KEGG enrichment analysis. The results of biological process (BP) analysis showed that the DEGs were mainly enriched in extracellular matrix tissues, regulation of DNA metabolism, response to transforming growth factor β, activation of protein kinase activity, and cell cycle G1/S phase transition. The results of cellular component (CC) analysis showed that the DEGs were mainly enriched in focal adhesions, cell-matrix adhesion junctions, extracellular matrix components, collagen-containing extracellular matrix, and fibrous collagen trimers. The results of molecular function (MF) analysis showed that the DEGs were mainly enriched in several functions including cell adhesion molecule binding, extracellular matrix structural components, growth factor binding, protein serine/threonine kinase activity, and collagen binding. The KEGG analysis showed that the DEGs were mainly enriched in focal adhesion, extracellular matrix-receptor interaction, PI3K-Akt signaling pathway, TGF-β signaling pathway, HCC, cell cycle, NOD-like receptor signaling pathway, PD-L1 expression and the PD-1 checkpoint pathway in cancer (Figure 3D). The results of Gene Set Enrichment Analysis (GESA) showed that CRGs were closely related to cancer pathways in HCC, including focal adhesions, cell cycle, T cell receptor signaling pathway, JAK STAT signaling pathway and MAPK signaling pathway. The activation of these pathways increased the risk of tumorigenesis and tumor progression (Figure 3E).

### Analysis of the correlation with immune infiltration and immune checkpoints

**FIGURE 4:**
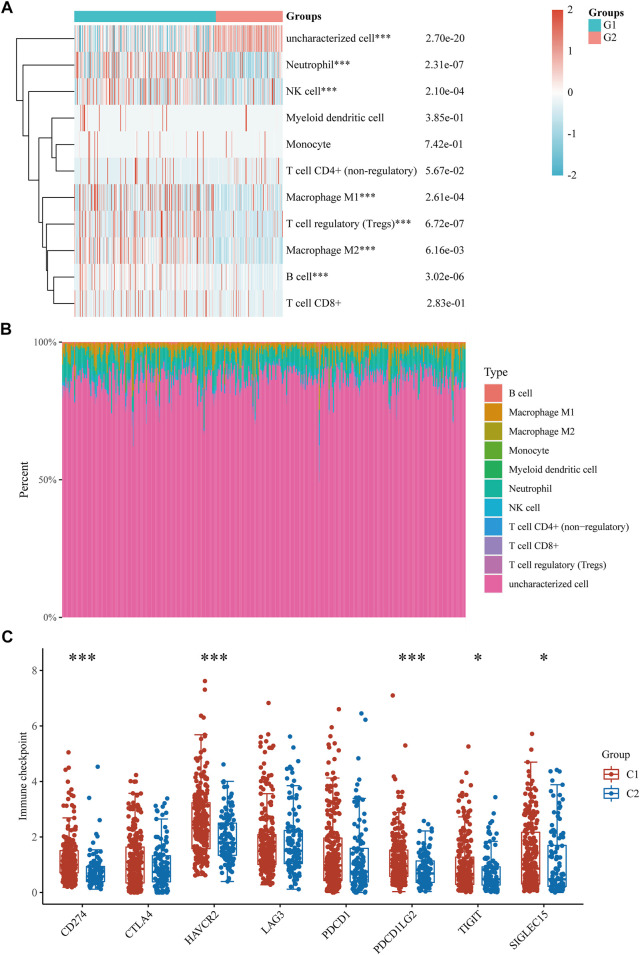
Immune infiltration estimated by quantTIseq algorithm and the expression distributions of 8 immune checkpoint-related genes in HCC subgroups. **(A)** Immune cell score heat map. **(B)** Proportions of 11 types of immune cells shown for each HCC patient by a histogram. **(C)** The expression distributions of 8 immune checkpoints-related genes in HCC subgroups. *p < 0.05; **p < 0.01; ***p < 0.001.

The TCGA-LIHC cohort was downloaded to explore the difference in immune cell infiltration between the C1 and C2 subtypes to explore the role of CRGs in the immune response in HCC. Six state-of-the-art algorithms were then integrated for a reliable immune score assessment. quantTIseq, EPIC, MCP-counter, CIBERSORT, xCell and TIMER showed the expression of CRGs was correlated with uncharacterized cell, Neutrophil, NK cell, Macrophage M1, T cell regulatory (Tregs), Macrophage M2, B cell (Figures 4A,B); Macrophage, uncharacterized cell, T cell CD4^+^, NK cell (Supplementary Figure S1A); NK cell, B cell, Myeloid dendritic cell, Monocyte, Macrophage/Monocyte, Neutrophil, Endothelial cell (Supplementary Figure S1B); Macrophage M2, T cell CD8^+^, T cell follicular helper, Mast cell activated, Neutrophil (Supplementary Figure S1C); Macrophage M2, Endothelial cell, Mast cell, T cell CD8^+^ naive, T cell CD4^+^ memory, Plasmacytoid dendritic cell, T cell CD4^+^ naive, Common lymphoid progenitor, T cell NK, T cell CD4^+^ Th1 (Supplementary Figure S1D); T cell CD4, Neutrophil, Macrophage, T cell CD8^+^, B cell, Myeloid dendritic cell (Supplementary Figure S1E) respectively. Finally, the difference between the two subgroups in the expression of eight immune checkpoint-related genes was explored, and the results showed that CD274 (*p* < 0.01), HAVCR (*p* < 0.01), PDCD1LG2 (*p* < 0.01), TIGIT (*p* < 0.05) and SIGLEC15 (*p* < 0.05) were highly expressed in the C1 subgroup than in the C2 subgroup (Figure 4C).

### DEGs and prognostic models

**FIGURE 5:**
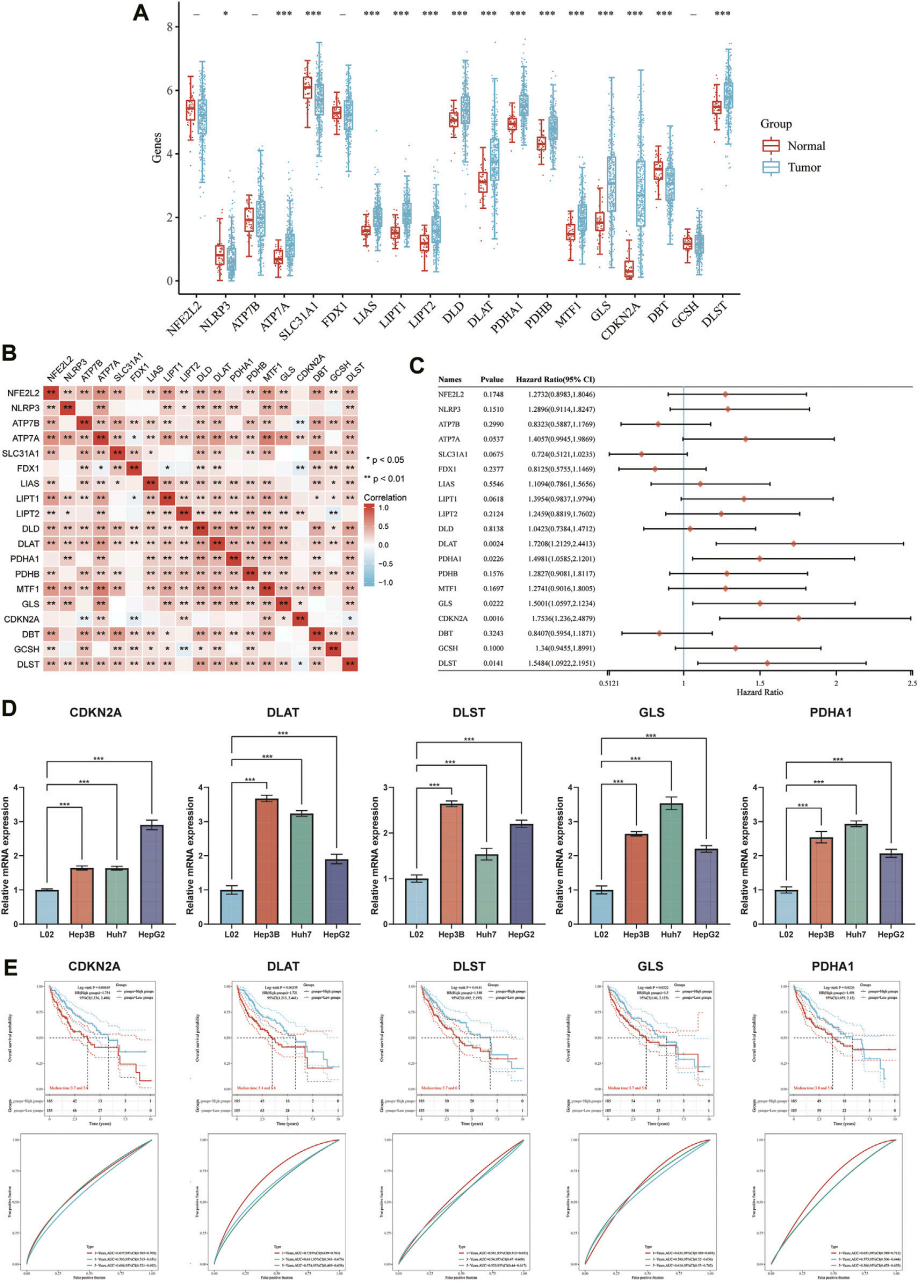
DEGs and prognostic models. **(A)** The expression of 19 CRGs in HCC and paracancerous tissues; the upper and lower ends of the box represent the interquartile range of the values. The line in the box represents the median. **(B)** Correlation between the expression of cuproptosis regulators. **(C)** Analysis of five prognostic CRGs from univariate Cox regression analysis plots. **(D)** mRNA expression of prognostic CRGs in HCC cell lines. **(E)** Prognostic value of five CRGs (*CDKN2A, DLAT, DLST, GLS,* and *PDHA1*) in HCC patients (OS curve of high/low expression group).

The expression of 19 CRGs in HCC and normal tissues was investigated using the TCGA dataset; among them, 15 CRGs were up- or downregulated. The expression of *ATP7A, SLC31A1, LIAS, LIPT1, LIPT2, DLD, DLAT, PDHA1, PDHB, MTF1, GLS, CDKN2A*, and *DLST* was upregulated, while *NLRP3* and *DBT* were downregulated in cancer tissues than in the normal tissues (Figure 5A). Most of the 19 CRGs in the HCC samples were positively correlated, while 6 pairs were negatively correlated, showing a strong correlation between them (Figure 5B). Univariate Cox regression analysis was then performed to screen CRGs with a prognostic value (Figure 5C). The mRNA expression of five prognostic CRGs was significantly upregulated (*p* < 0.001) in HCC cell lines (Hep3B, Huh7 and HepG2) compared to their expression in the hepatocyte cell line (L02) (Figure 5D). Finally, five genes with a prognostic value were identified from the results. The Kaplan-Meier survival curves are shown in Figure 5E. HCC patients had a lower survival rate when *CDKN2A* (HR = 1.75, *p* = 0.002), *DLAT* (HR = 1.7208, *p* = 0.0024), *DLST* (HR = 1.5484, *p* = 0.0141), *GLS* (HR = 1.5001, *p* = 0.0222) and *PDHA1* (HR = 1.4981, *p* = 0.0226) were highly expressed.

### Construction of a prognostic CRG model

**FIGURE 6:**
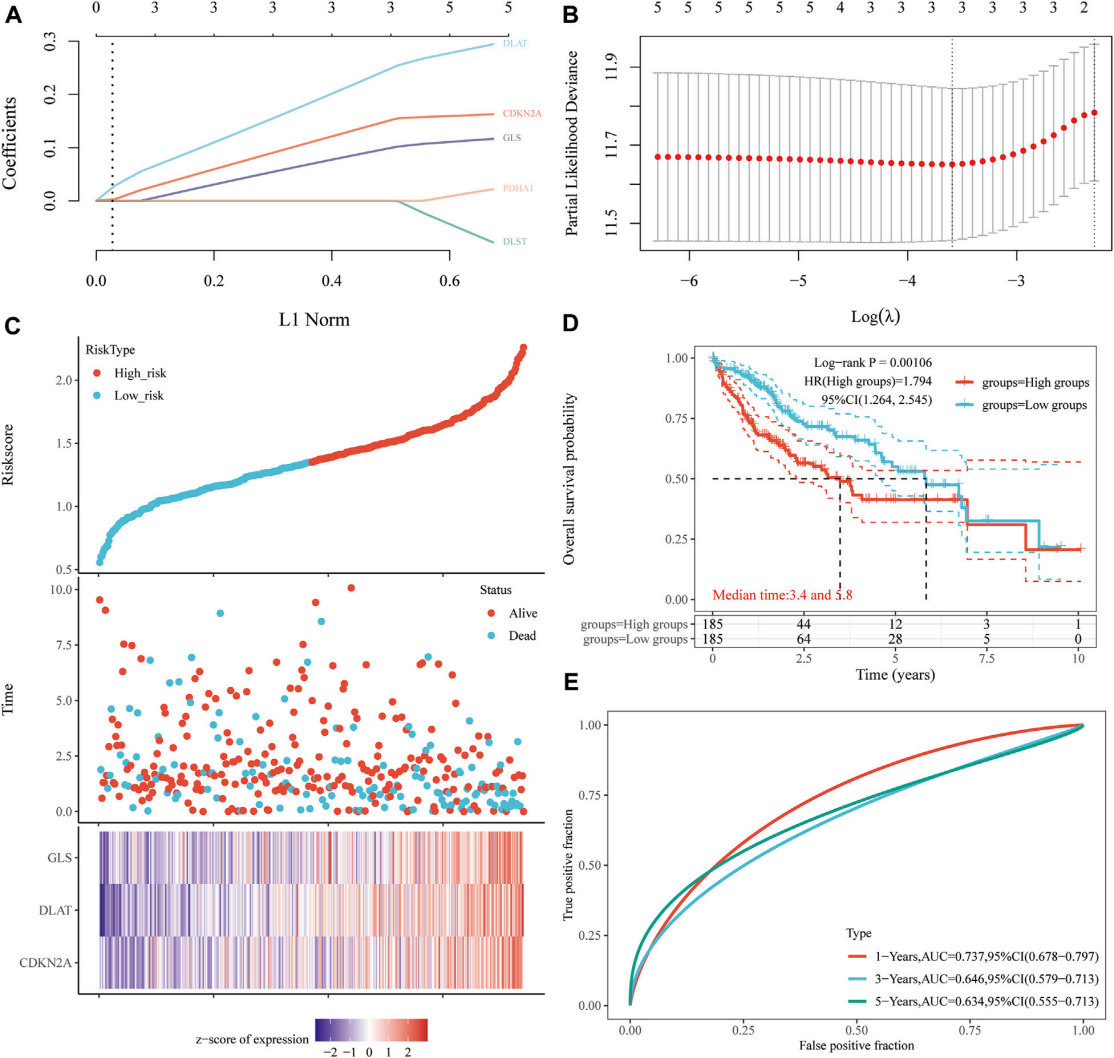
Construction of a prognostic CRG model. **(A)** LASSO coefficient profiles of prognostic CRGs, **(B)** Plots of the ten-fold cross-validation error rates. **(C)** Distribution of the risk score, survival status, and the expression of prognostic CRGs in HCC. **(D, E)** OS curves of HCC patients in the high-/low-risk group and the ROC curve for measuring the predictive value.

A prognostic gene model based on prognostic CRGs was constructed by LASSO Cox regression analysis (Figures 6A,B). The risk score was calculated as follows: (0.1242)*CDKN2A+ (0.2058)*DLAT + (0.0795)*GLS. HCC patients were divided into two groups according to the risk score distribution, survival status, and expression of *GLS, DLAT,* and *CDKN2A* (Figures 6C,D). Patients had an increased risk of death and decreased survival time as the risk scores increased (Figure 6C). The Kaplan-Meier curves showed that HCC patients with high-risk scores had a lower probability of OS than patients with low-risk scores (median time = 3.4 years vs*.* 5.8 years, *p* = 0.00106) (Figure 6D). The area under the ROC curve (AUC) in the 1-year, 3-year and 5-year ROC curves was 0.737, 0.646 and 0.634, respectively (Figure 6E). The results of our constructed cuproptosis-related risk profile showed a significant association with survival in patients with HCC.

### Building of a predictive nomogram

**FIGURE 7:**
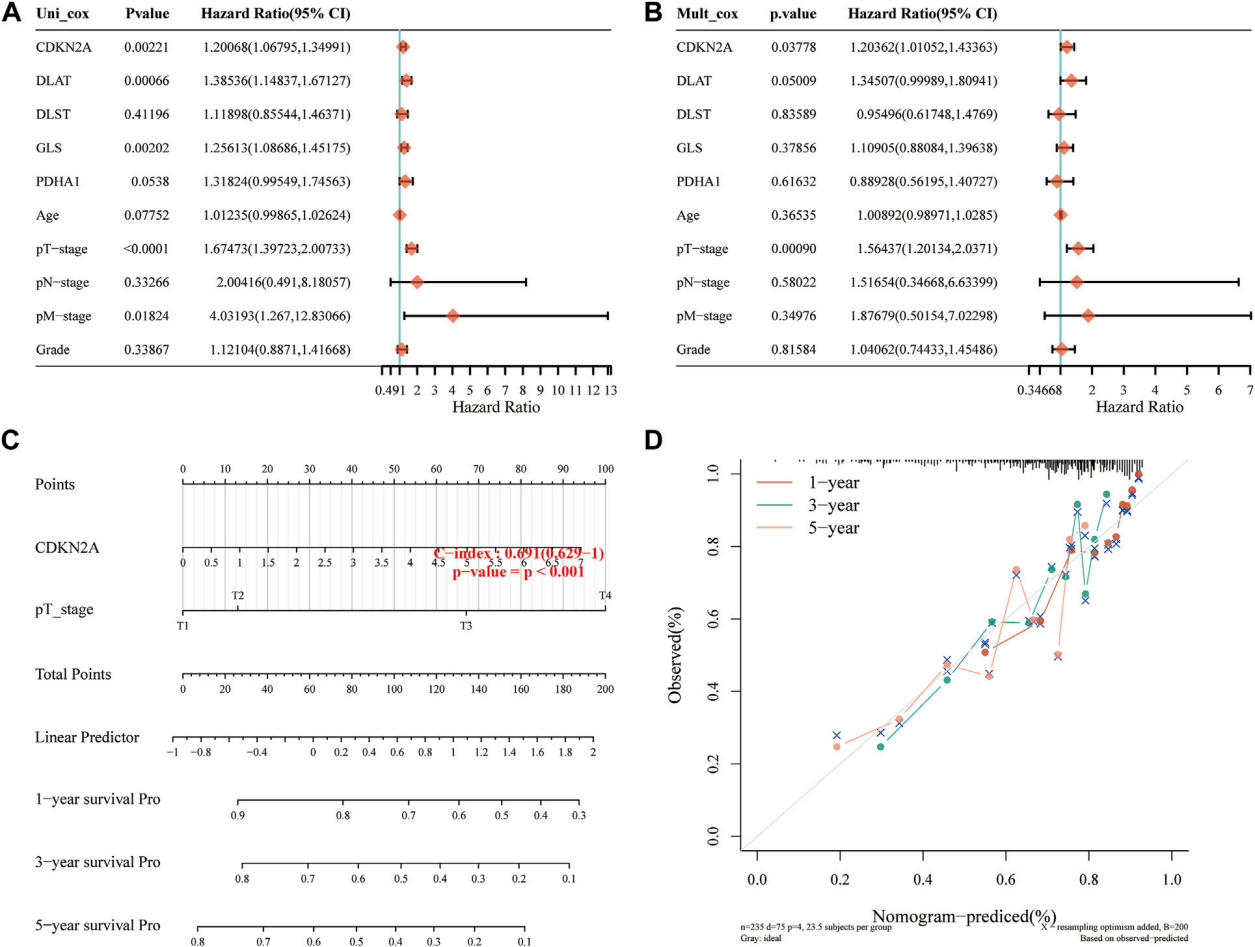
Construction of a predictive nomogram. **(A, B)** Hazard ratios and *p*-value of the constituents involved in univariate and multivariate Cox regression analysis considering the clinical information and prognostic CRGs in HCC. **(C)** Nomogram to predict the 1-year, 3-year and 5-year OS rate of HCC patients. **(D)** Calibration curve for the OS nomogram model in the discovery group. The dashed diagonal line represents the ideal nomogram. OS, overall survival.

Considering the clinicopathologic features and prognostic CRGs, a predictive nomogram was also built to predict the survival probability. The results of the univariate and multivariate analysis showed that *CDKN2A* expression and pT stage were independent factors affecting the prognosis of HCC patients (Figures 7A,B; Supplementary Figure S2). The predictive nomogram suggested that the 3-year and 5-year OS rates and PFS rates were predicted enough accurately compared with an ideal model in the entire cohort (Figures 7C,D; Supplementary Figure S2).

### Correlation between prognostic CRGs and pathologic stage in HCC

Based on the above analysis, the correlation between the expression of CRGs and the pathological stage of HCC was analyzed by Gene Expression Profiling Interactive Analysis (GEPIA) 2 database. The results showed that the expression of *CDKN2A, DLAT, GLS* and *PDHA1* were significantly correlated with the pathological stage of HCC (*p* < 0.05), while the correlation between *DLST* and the pathological stage was not significant (Supplementary Figure S3). The analysis of UALCAN database revealed that the expression of prognostic CRGs in HCC patients in stages 1–3 was significantly upregulated compared with their expression in the normal group. The analysis of TISIDB database revealed that the expression of *CDKN2A, DLST,* and *GLS* in different stages and scores of HCC patients was significantly different, while *DLAT* and *PDHA1* was slightly changed although not significantly (Supplementary Figure S3).

### Pathological expression of CRGs in HCC tissues and normal livers

**FIGURE 8:**
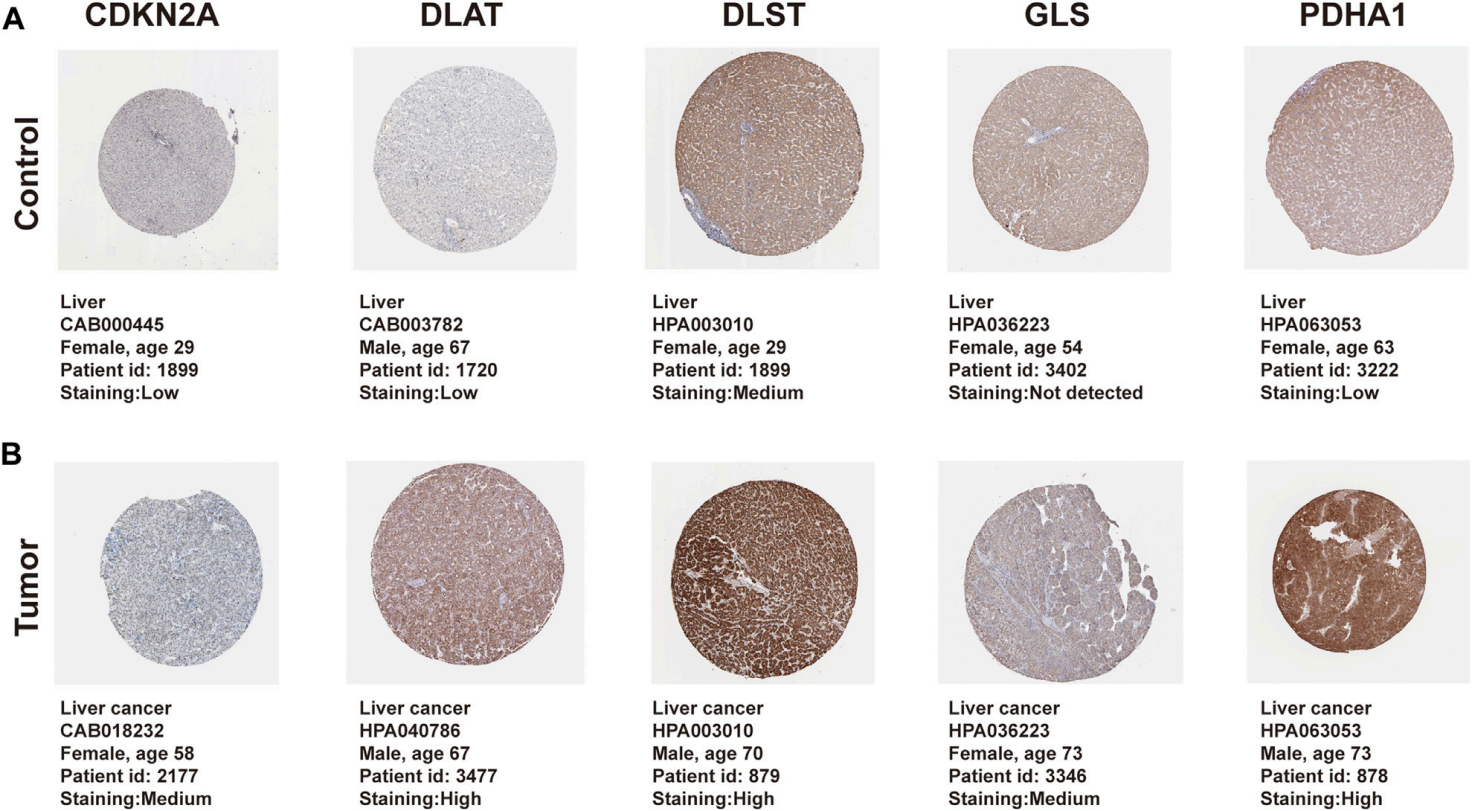
Protein differential expression of prognostic CRGs in normal liver and HCC tissue. **(A)** Protein expression of prognostic CRGs in normal liver. **(B)** Protein expression of prognostic CRGs in HCC tissue. Immunohistochemical data were obtained from the Human Protein Atlas (×200 magnification).

The protein expression of CRGs in 371 HCC and adjacent tissues was measured using the Human Protein Atlas to determine the difference in protein expression of the prognostic CRGs in HCC tissues and normal liver. The results of the immunohistochemical staining showed that the prognostic CRGs were moderately or highly expressed in HCC tissues but were low in paracancerous tissues (Figures 8A,B).

### Association of genetic mutations in CRGs with survival and clinical outcomes of HCC patients

**FIGURE 9:**
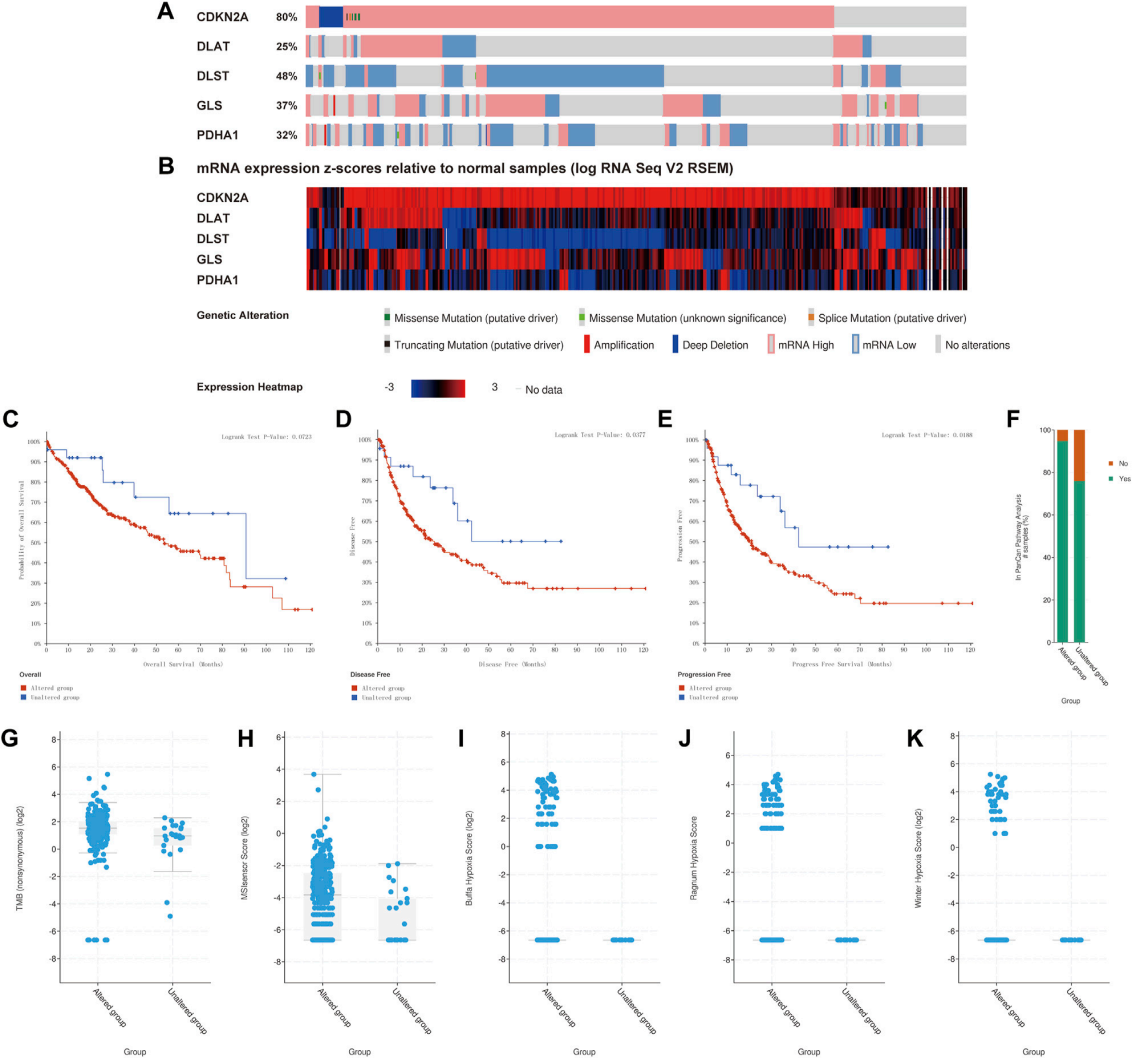
Association of gene mutations in prognostic CRGs with survival and clinical outcomes of HCC patients (cBioPortal). **(A)** Mutation rate of prognostic CRGs in HCC patients. **(B)** mRNA expression z-score of prognostic CRGs in HCC tissue samples relative to normal samples. **(C–E)** CRG mutations in liver cancer tissues associated with shorter OS, DFS, and PFS in HCC patients. **(F–K)** CRG mutations in HCC tissues upregulated cancer progression-related scores, including In PanCan Pathway Analysis **(F)**, TMB **(G)**, MSIsenso Score **(H)**, Buffa Hypoxia Score **(I)**, Ragnum Hypoxia Score **(J)** and Winter Hypoxia Score **(K)**.

The genetic alteration of the prognostic CRGs were analyzed using the cBio Cancer Genomics Portal (cBioPortal) online tool for HCC. *CDKN2A, DLAT, DLST, GLS*, and *PDHA1* were altered in 80%, 25%, 48%, 37%, and 32% of the 347 samples of the 372 sequenced patients, as shown in Figure 9A. The mRNA expression z-scores of the prognostic CRGs relative to normal samples are shown in Figure 9B. The survival analysis results showed that the genetic alteration in prognostic CRGs was significantly associated with shorter disease free survival (DFS) (*p* = 0.0377, Figure 9D) and PFS (Figure 9E, *p* = 0.018) of HCC patients. However, these two groups have little correlation with OS (*p* = 0.0723, Figure 9C). In addition, the clinical analysis showed that HCC tissues with prognostic CRG mutations upregulated the expression of cancer-related signaling pathways (*p* = 1.051 × 10^−3^, Figure 9F), tumor burden mutation (TMB) (*p* = 1.490 × 10^−3^, Figure 9G), MSIsensor Score (*p* = 1.426 × 10^−3^, Figure 9H), as well as hypoxia scores, including Buffa Hypoxia Score (*p* = 2.329 × 10^−3^, Figure 9I), Ragnum Hypoxia Score (*p* = 3.249 × 10^−5^, Figure 9J) and Winter Hypoxia Score (*p* = 9.727 × 10^−4^, Figure 9K). Therefore, the mutation of prognostic CRGs in HCC tissues significantly affected the prognosis of HCC patients.

### Prognostic CRGs interfere with immune cell infiltration and immune checkpoint expression in HCC

**FIGURE 10:**
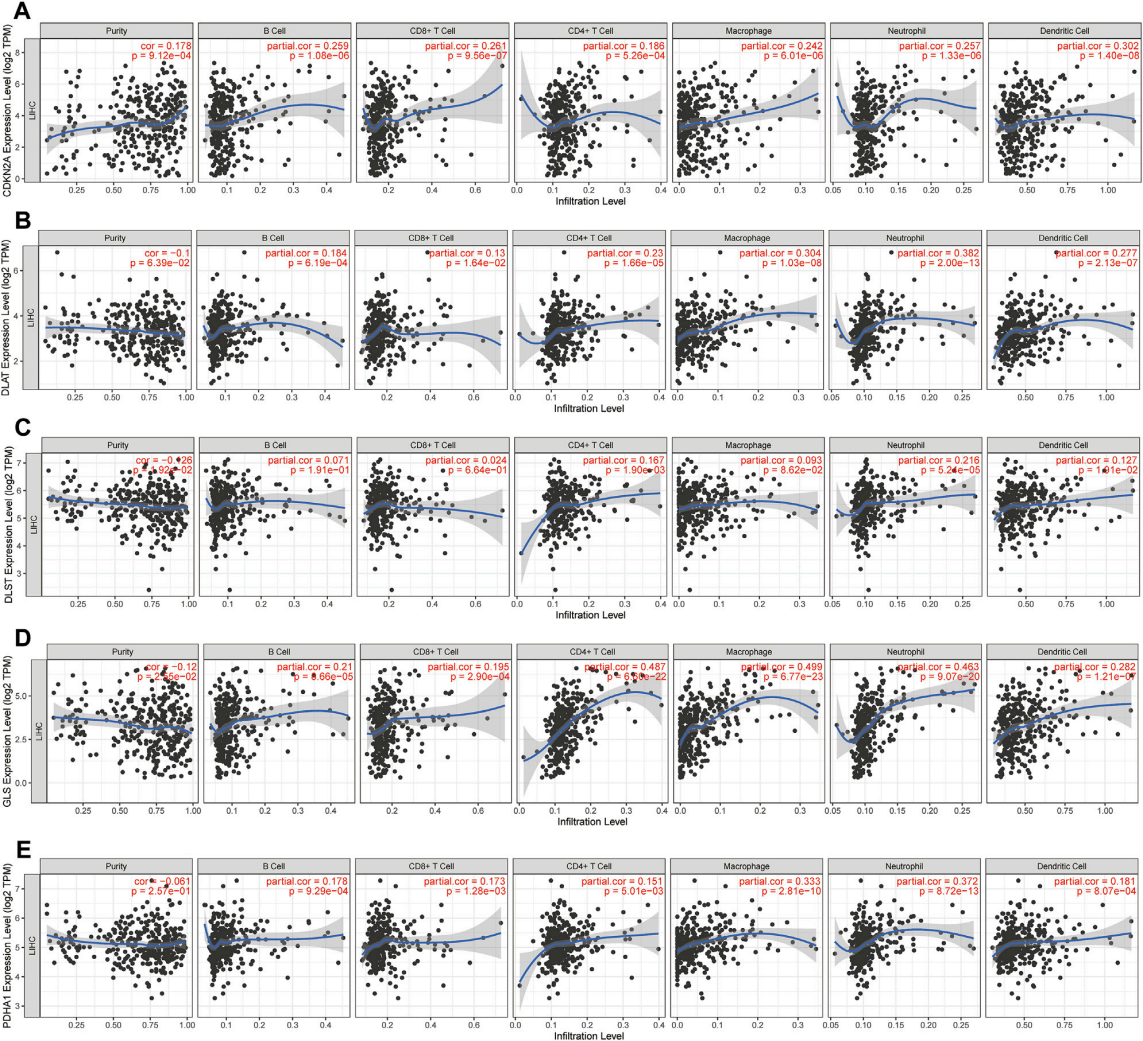
Prognostic CRGs involved in immune infiltration in HCC. **(A-E)** Association between immune cell abundance in HCC and prognostic CRGs (TIMER database).

The above results demonstrated that CRGs play a crucial role in the development of tumor immune microenvironment in HCC. The present study further investigated the correlation between the expression of CRGs (*CDKN2A, DLAT, DLST, GLS*, and *PDHA1*) and immune infiltration in HCC using the TIMER database and TCGA database. TIMER data showed that *CDKN2A, DLAT, GLS* and *PDHA1* were strongly and positively correlated with tumor purity, B cells, CD4^+^ T cells, CD8^+^ T cells, neutrophils, macrophages, and dendritic cells, while *DLST* was only positively correlated with tumor purity, CD4^+^ T cells, neutrophils, and dendritic cells (Figures 10A–E). A total of 24 immune cell types infiltrating HCC were next identified using single-sample GSEA, followed by Spearman analysis to investigate the association between prognostic CRGs and immune cell infiltration. The high expression of prognostic CRGs was positively correlated with several cell types, including T helper cells, Th1 cells, TFH, and aDC, while negatively correlated with cytotoxic cells, DC, and pDC, as shown in Supplementary Figure S4. In addition, the correlation between prognostic CRG expression and important immune checkpoints (CD274, PD-L1 and CTLA4) was further investigated. The results showed that *CDKN2A* was significantly and positively correlated with CD274 (*p* = 2.41 × 10^−05^, cor = 0.217), PD-L1 (*p* = 1.24 × 10^−03^, cor = 0.167) and CTLA-4 (*p* = 3.62 × 10^−05^, cor = 0.213), and *GLS* was also positively correlated with CD274 (*p* = 3.02 × 10^−09^, cor = 0.302), PD-L1 (*p* = 4.25e−07, cor = 0.259) and CTLA-4 (*p* = 8 × 10^−05^, cor = 0.203), while *DLAT* (*p* = 3.27e−13, cor = 0.366), *DLST* (*p* = 3.71e−10, cor = 0.318) and *PDHA1* (*p* = 1.77e−07, cor = 0.267) were significantly and positively correlated only with CD274 (Supplementary Figure S5). These results indicated a significant correlation between prognostic CRGs and tumor immune infiltration and showed that prognostic CRGs might be predictive markers of anti-CD274 therapy.

### TMB, microsatellite instability and drug sensitivity analysis

**FIGURE 11:**
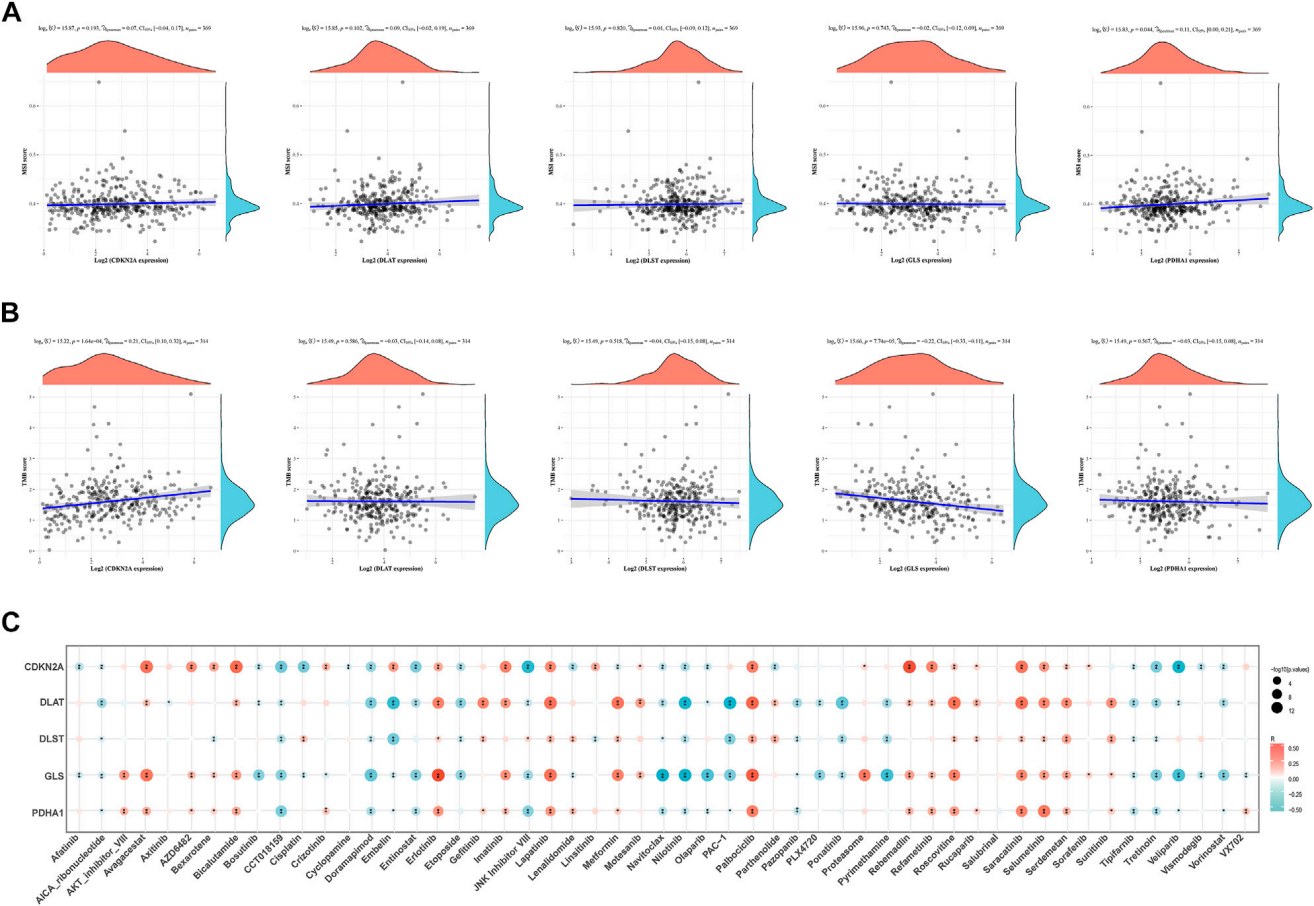
TMB, MSI and drug sensitivity. **(A)** Correlation between the expression of prognostic CRGs and MSI in HCC. **(B)** Correlation between prognostic CRGs and TMB in HCC. **(C)** Correlation between prognostic CRGs and antitumor drugs in HCC. TMB, tumor mutational burden; MSI, microsatellite instability.

Microsatellite instability (MSI) and TMB can be used as indicators to predict the response of various tumors to immunotherapy (Rizzo et al., 2021). The above results showed that the expression of CRGs was closely related to tumor immune cell infiltration in HCC. Next, the association of prognostic CRGs with TMB and MSI in HCC was analyzed to determine whether CRGs can be considered as biomarkers for immunotherapy. The results showed that *PDHA1* was positively correlated with MSI (*p* = 0.044) (Figure 11A). Furthermore, *CDKN2A* (*p* = 1.64 × 10^−04^) was positively correlated with TMB, and *GLS* (*p* = 7.74 × 10^−05^) and negatively correlated with TMB (Figure 11B). Finally, the data on the gene expression profiles of cancer cell lines were integrated in the Genomics of Drug Sensitivity in Cancer (GDSC) database for drug sensitivity to fully explore the potential value of *CDKN2A, DLAT, DLST, GLS*, and *PDHA1* genes as novel therapeutic targets. A Pearson correlation analysis of the data was performed, which showed that the expression of *CDKN2A, DLAT, DLST, GLS*, and *PDHA1* was positively correlated with Saracatinib, Selumetinib, Serdemetan, Rucaparib, Roscovitine, Refametinib, Proteasome, Palbociclib, Motesanib, Lapatinib, Imatinib and Erlotinib, but negatively correlated with Tretinoin, Tipifarnib, Nilotinib, Navitoclax, Doramapimod, CCT018159 and AICA_ribonucleotide (Figure 11C).

### Correlation between CRGs and m6A methylation-related genes in HCC

**FIGURE 12:**
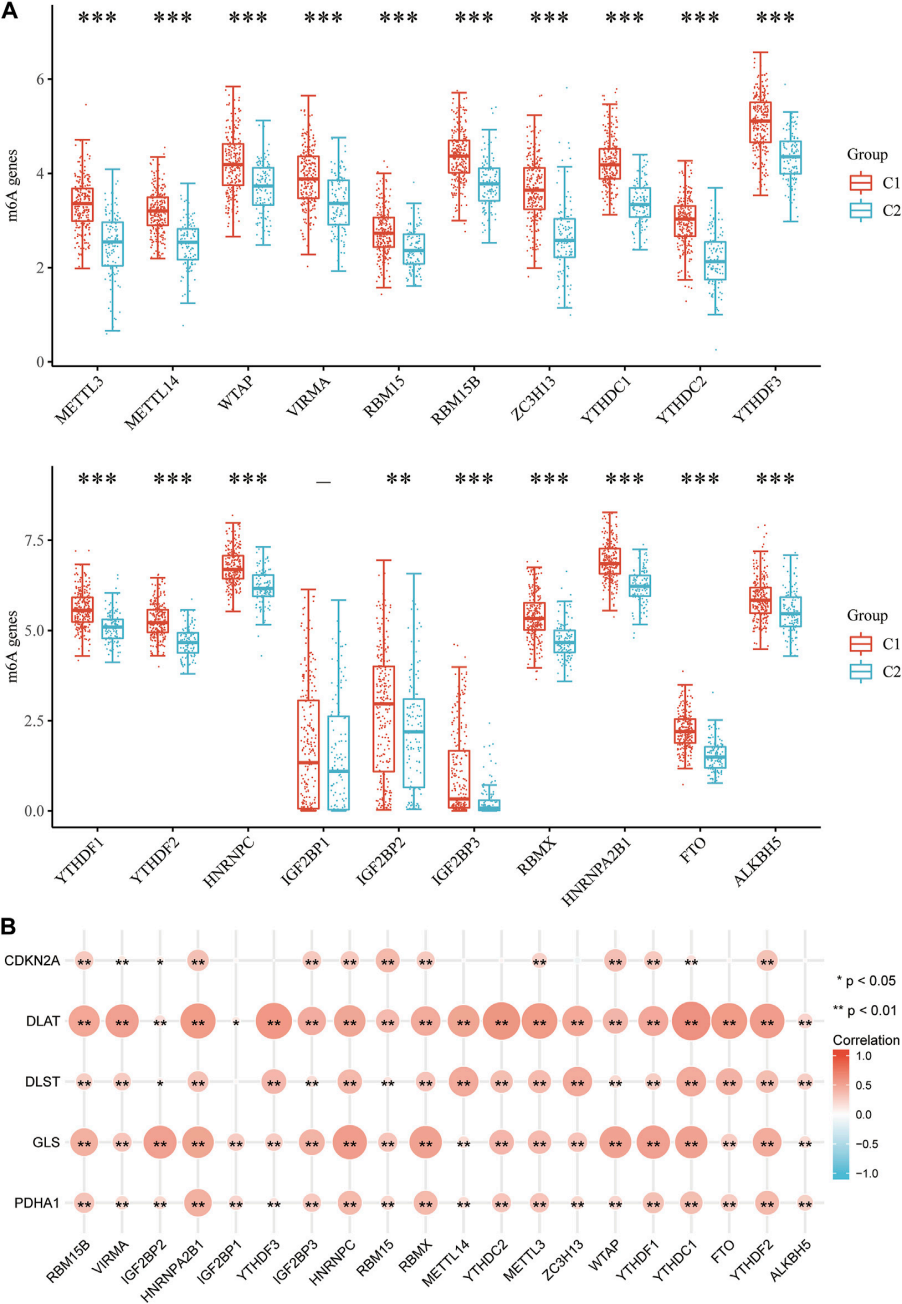
Correlation of CRG expression and m6A-related genes in HCC. **(A)** Differential expression of m6A-related genes between the C1 and C2 subgroups in HCC. **(B)** Analysis of the relationship between prognostic CRGs and m6A-related genes by TCGA-LIHC cohort.

It is well known that m6A methylation modification affects gene expression by regulating RNA metabolism and plays an important role in the occurrence and development of malignant tumors. The potential correlation of CRGs with m6A modification in HCC was evaluated, and the differential expression of 20 m6A-related genes (RBM15B, VIRMA, IGF2BP29, HNRNPA2B1, IGF2BP1, YTHDF3, IGF2BP3, HNRNPC4, RBM15, RBMX, METTL14, YTHDC2, METTL3, ZC3H13, WTAP, YTHDF1, YTHDC1, FTO, YTHDF2) were found between the C1 and C2 subgroups. The results showed that except *IGF2BP1*, other m6A-related genes were significantly different between the two groups (*p* < 0.01, Figure 12A). The C1 group with higher expression of CRGs has also higher expression of m6A methylation-related genes. In addition, the correlation between prognostic CRGs and m6A-related genes was analyzed by the TCGA dataset, and the results showed a significant positive correlation between prognostic CRGs and m6A-related genes (Figure 12B). The above results suggested that CRGs were closely related to m6A modification in HCC.

### Single-cell RNA data analysis

**FIGURE 13:**
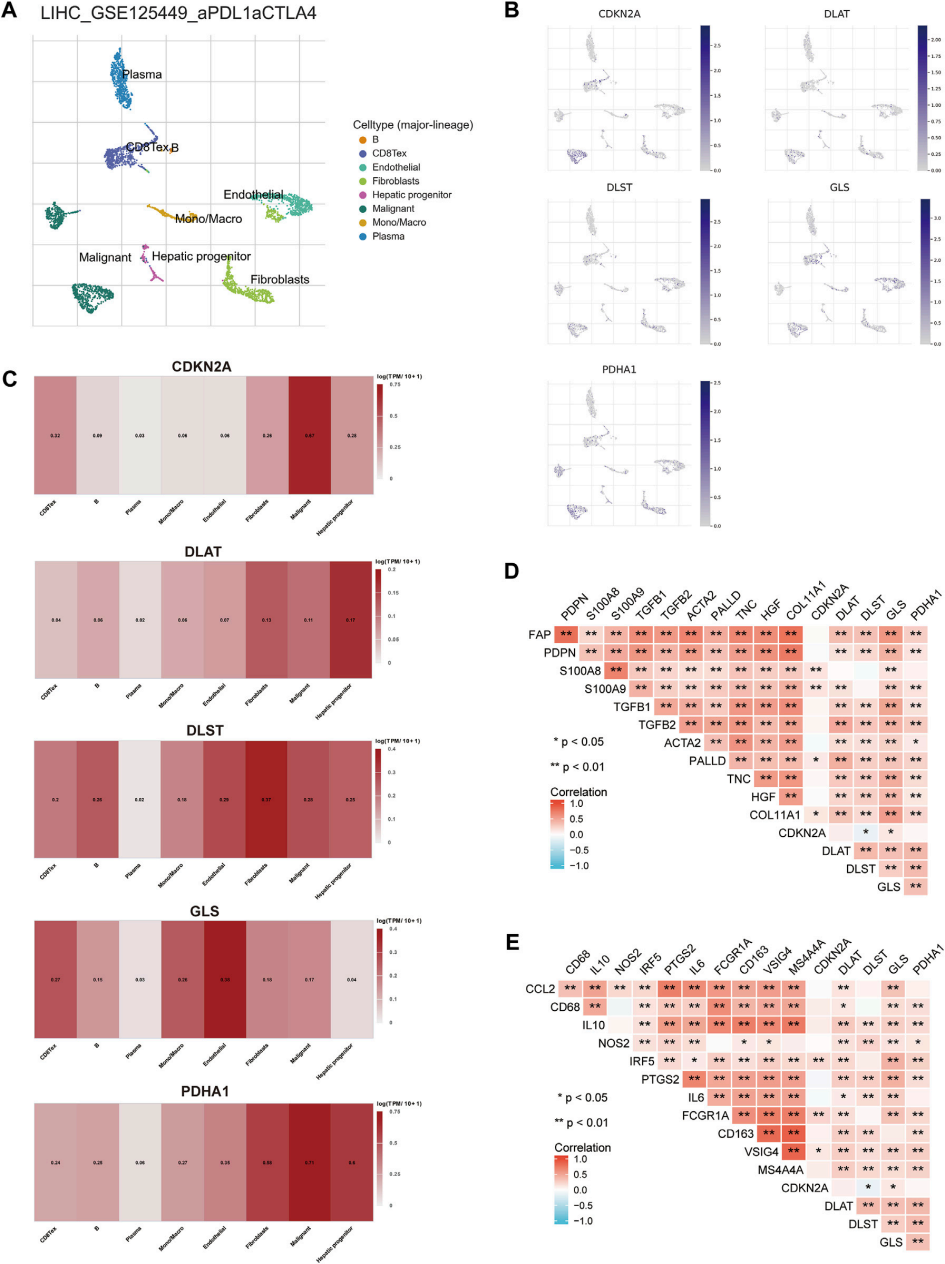
Expression of CRGs in different immune cell types in HCC. **(A)** Cluster diagram of cell types in scRNA-seq data. t-SNE plot showing the expression of different immune cells (LIHC_GSE125449_aPDL1aCTLA4) in liver cancer tissue. **(B, C)** Characteristic maps of prognostic CRGs obtained from scRNA-seq data. **(D)** Correlation between prognostic CRGs and CAF-related markers. **(E)** Correlation between prognostic CRGs and TAM-related markers. **p < 0.01; CAF, cancer-associated fibroblasts; TAM, tumor-associated macrophage.

The TME is composed of extracellular matrix, cancer associated fibroblasts (CAFs), myofibroblasts, immune cells and other factors. The prognostic CRGs were assessed at a single-cell level expression by Tumor Immunity Single Cell Center (TISCH, http://tisch.comp-genomics.org/) to examine the relationship between prognostic CRGs and the infiltration of immune cells, stromal cells, and malignant cells. The HCC single-cell GSE dataset was explored (LIHC_GSE125449_aPDL1aCTLA4). T cells, B cells, plasma cells, macrophages, endothelial cells, fibroblasts, malignant cells, and hepatic progenitors were annotated by single-cell RNA sequencing analysis (Figure 13A). The results showed that *CDKN2A, DLAT, DLST, GLS*, and *PDHA1* were correlated in single cell subsets such as immune cells, stromal cells, and malignant cells, and were significantly expressed in macrophages, fibroblasts, endothelial cells, and hepatic progenitor cells (Figures 13B,C). CSFs and tumor-associated macrophages (TAMs) play an important role in the occurrence and development of cancer (Rizzo et al., 2021). Therefore, the association between prognostic CRGs and biomarkers associated with CAFs and TAMs was further explored. The results showed that prognostic CRGs were significantly and positively correlated with CAF markers such as FAP, PDPN, S100A8, S100A9, TGFB1, TGFB2, ACTA2, PALLD, TNC, and COL11A1 (Figure 13D). Prognostic CRGs were also highly correlated with TAM-related markers such as CCL2, CD68, and IL 10 (Figure 13E).

### Prediction and validation of upstream key miRNAs

**FIGURE 14:**
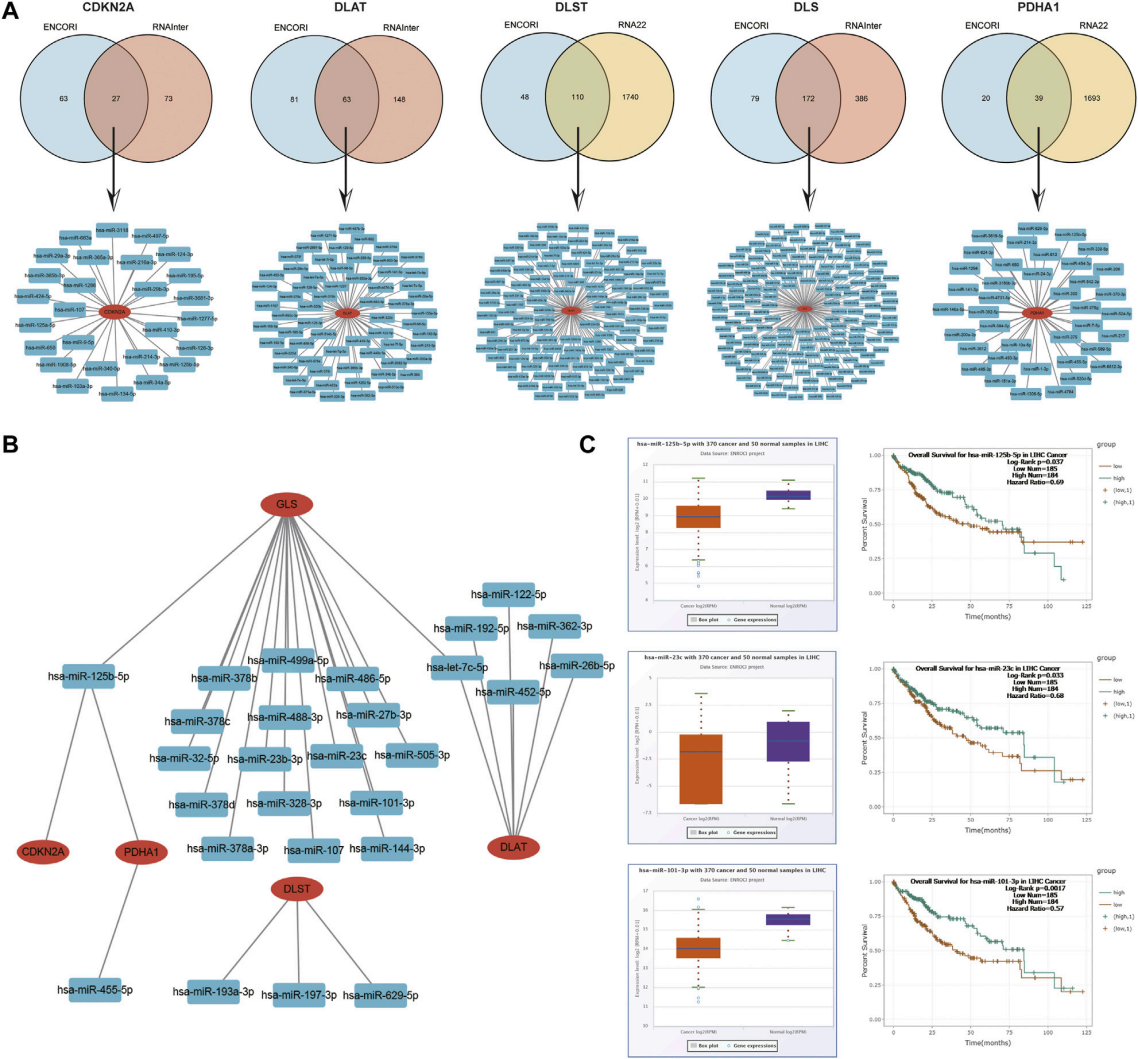
Identification of the potential miRNAs related to the prognosis of HCC. **(A)** Potential predicted miRNAs associated with CRGs by ENCORI, RNAInter and RNA22 databases. **(B)** Potential mRNA-miRNA gene networks constructed using Cytoscape software. **(C)** Expression and prognostic value of miRNAs (hsa-miR-125b-5p, hsa-miR-101-3p and hsa-miR-23c).

Next, the upstream regulatory miRNAs of prognostic CRGs were predicted using a comprehensive miRNA-related database. Firstly, 27 pairs of *CDKN2A*-miRNAs, 63 pairs of *DLAT*-miRNAs and 172 pairs of *GLS*-miRNAs were obtained by the intersection of ENCORI and RNAInter databases. A total of 110 pairs of *DLST*-miRNAs and 39 pairs of *PDHA1*-miRNAs were obtained by the intersection of ENCORI databases and RNA22 databases (Figure 14A). Then, according to the classical mechanism of miRNAs negatively regulating mRNA expression, a negative correlation between the mRNA and the predicted miRNA was expected. The ENCORI database was used to screen the correlation, prognosis and expression of these candidate miRNAs in HCC. Among these miRNA-mRNA interactions, the results showed that 1 pair of miRNA-*CDKN2A*, 6 pairs of miRNAs-*DLAT*, 3 pairs of miRNAs-*DLST*, 18 pairs of miRNAs-*GLS*, and 2 pairs of miRNAs-*PDHA1* were significantly and negatively correlated (Figure 14B; Supplementary Figure S6). Theoretically, miRNAs that bind to prognostic CRGs should be downregulated and indicate poor prognosis in HCC. Therefore, the prognostic role and expression of these potential miRNAs in HCC were further confirmed using the ENCORI database. The results showed that only the low expressed hsa-miR-125b-5p, hsa-miR-101-3p and hsa-miR-23c corresponded to a poor prognosis, and hsa-miR-125b-5p, hsa-miR-101-3p and hsa-miR-23c were significantly lower in HCC tissues than in normal tissues (Figure 14C). All these results indicated that *CDKN2A*-hsa-miR-125b-5p, *GLS*-hsa-miR-125b-5p, *GLS*-hsa-miR-101–3, *GLS*-hsa-miR-23c and *PDHA1*-hsa-miR-125b-5p might represent key pathways that mediated the occurrence and development of HCC and were related to the prognosis of patients.

### Prediction and validation of key lncRNAs binding to potential miRNAs

**FIGURE 15:**
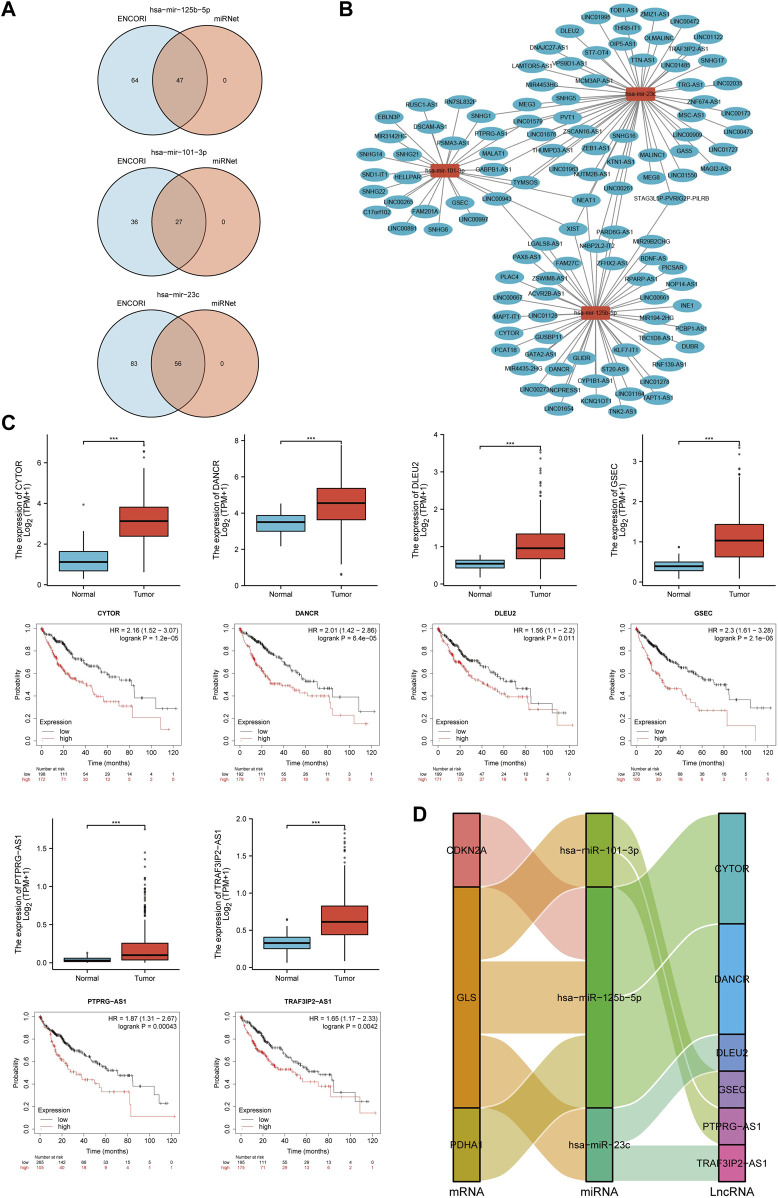
Screening of the regulatory axis of lncRNA-miRNA-CRGs in HCC. **(A)** Potential lncRNAs associated with hsa-miR-125b-5p, hsa-miR-101-3p and hsa-miR-23c predicted by miRNet and ENCORI databases. **(B)** Potential miRNA-lncRNA network constructed using Cytoscape software. **(C)** Expression and prognostic value of six potential lncRNAs in HCC. **(D)** lncRNA-miRNA-mRNA triple regulatory network affecting the prognosis of HCC.

Previous studies showed that lncRNAs bind to miRNAs and mediate the expression of target genes, thereby exerting biological roles (Militello et al., 2017; Zhang and Lou, 2020). Based on the above results, lncRNAs upstream of miRNAs were also predicted to construct the miRNA-lncRNA axis. The lncRNAs potentially binding to hsa-miR-101-3p, hsa-miR-125b-5p and hsa-miR-23c were predicted by the intersection of ENCORI and miRNet databases, and the results revealed 27 lncRNAs targeting hsa-miR-101-3p, 47 lncRNAs targeting hsa-miR-125b-5p and 56 lncRNAs targeting hsa-miR-23c (Figure 15A). A miRNA-lncRNA regulatory network was established by Cytoscape software for a better visualization (Figure 15B). According to the ceRNA hypothesis, lncRNAs can increase mRNA expression by competitively binding to miRNAs. Therefore, lncRNAs were negatively correlated with miRNAs or positively correlated with mRNAs. The correlation between lncRNAs and hsa-miR-101-3p, hsa-miR-125b-5p and hsa-miR-23c was detected by the ENCORI database, and the results showed that 14 lncRNAs were significantly associated with hsa-miR-101-3p and *GLS*, 9 lncRNAs were significantly associated with hsa-miR-125b-5p and *CDKN2A*, 10 lncRNAs were significantly associated with hsa-miR-125b-5p and *GLS*, 4 lncRNAs were significantly associated with hsa-miR-125b-5p and *PDHA1*, and 12 lncRNAs were significantly associated with hsa-miR-23c and *GLS* (Supplementary Tables S2–6; Supplementary Figure S7). Subsequently, the prognostic value of lncRNAs in HCC was assessed by the Kaplan-Meier plotter, and the expression of these lncRNAs in HCC were detected using the TCGA-LIHC cohort. The results in combination with the survival analysis and expression analysis, showed that GSEC, PTPRG-AS1, CYTOR, DANCR, TRAF3IP2-AS1 and DLEU2 were significantly upregulated in HCC, and their upregulation was associated with poor prognosis in HCC patients (Figure 15C). Finally, a key mRNA-miRNA-lncRNA triple regulatory network associated with HCC prognosis was established, which included 3 mRNAs (*CDKN2A*, *GLS*, and *PDHA1*), 3 miRNAs (hsa-miR-125b-5p, hsa-miR-101-3p and hsa-miR-23c) and 6 lncRNAs (GSEC, PTPRG-AS1, CYTOR, DANCR, TRAF3IP2-AS1 and DLEU2). Eight lncRNA-miRNA-mRNA networks were also constructed, including lncRNA CYTOR/miR-125-5p/CDKN2A, lncRNA CYTOR/miR-125-5p/GLS, lncRNA DANC R/miR-125-5p/GLS, lncRNA DANCR/miR-125-5p/PDHA1, lncRNA DLEU2/miR-23c/GLS, lncRNA GSEC/miR-101-3p/GLS, lncRNA PTPRG-AS1/miR-101-3p/GLS and lncRNA TRAF3IP2-AS1/miR-23c/GLS regulatory axes. (Figure 15D).

## Discussion

Cuproptosis is a novel cell death mechanism that is characterized by cell death induced by copper, which targets proteins of the fatty acylated tricarboxylic acid cycle (Tsvetkov et al., 2022). Wilson’s disease is a condition caused by copper accumulation. Previous studies showed that humans or animal models with Wilson’s disease have an increased incidence of HCC, indicating that the accumulation of copper promotes the malignant transformation, but the mechanism is not yet clear (Czlonkowska et al., 2018). CRGs play a crucial role in the development of kidney cancer (Bian et al., 2022). However, the role of CRGs in HCC has not yet been elucidated. Therefore, a bioinformatic analysis of public sequencing data of CRGs in HCC was performed in this work to gain a deeper understanding of CRGs expression, prognosis and their potential biological functions in HCC and RT-qPCR was used for experimental validation to guide future research.

The results showed that these 19 CRGs were mainly involved in extracellular matrix organization, regulation of DNA metabolism, cell-matrix adhesion junction, extracellular matrix components, cell adhesion molecule binding, collagen binding and other biological functions in HCC, and they were closely related to focal adhesion, extracellular matrix-receptor interaction, HCC, cell cycle, PD-L1 expression, PD-1 checkpoint, PI3K-Akt, TGF-β, NOD-like receptor and other signaling pathways. The results of GSEA enrichment analysis showed that the potential biological processes and pathways involved in CRGs in HCC included focal adhesion, cell cycle, T cell receptor, JAK STAT, MAPK and other signaling pathways. These pathways are closely related to tumorigenesis and tumor progression. The adhesion of cancer cells to the extracellular matrix promotes the resistance of cancer cells to chemotherapeutic drugs, thereby promoting the occurrence and development of tumors (Eke and Cordes, 2015). Signaling proteins at focal adhesions include kinases such as focal adhesion kinase, integrin-linked kinase, and phosphatases. Focal adhesion kinase is a cytoplasmic tyrosine kinase identified as a key mediator of integrin signaling (Seong et al., 2013). It plays an important role in tumor progression and metastasis by regulating cancer cell functions such as migration, invasion, and epithelial-mesenchymal transition, as well as affecting the pericancer microenvironment such as angiogenesis (Zhao and Guan, 2009). The focal adhesion kinase inhibitor BI853520 inhibits the proliferation, migration, invasion and epithelial-mesenchymal transition of cancer cells by affecting the PI3K/AKT/mTOR signaling pathway (Zhang et al., 2020; Li et al., 2021). Our results suggested that CRGs may affect the occurrence and progression of HCC by affecting focal adhesion and various cancer-related signaling pathways.

According to our results the expression of most CRGs in HCC tissues was significantly higher than that in the adjacent tissues. Quantitative RT-PCR results also confirmed that The expression of CDKN2A, DLAT, DLST, GLS, and PDHA1 was higher in various HCC cell lines than in the hepatocyte cell line (L02). The results of prognostic analysis showed that HCC patients with higher expression of CDKN2A, DLAT, DLST, GLS, and PDHA1 had shorter OS, indicating that prognostic CRGs promoted the progression of HCC. CDKN2A is highly expressed in various cancer tissues such as liver cancer and kidney cancer and plays an important prognostic role in many cancers (Ai et al., 2003; Zeng et al., 2018; Christodoulou et al., 2020; Ji et al., 2020; Xande et al., 2020). DLST is an E2 component of the α-ketoglutarate (αKG) dehydrogenase complex, which governs the entry of glutamine into the TCA for oxidative decarboxylation, thus promotes neuroblastoma aggression (Anderson et al., 2021). Over expression of glycolytic gene DLAT, which promoted glycolysis but suppressed acetyl-CoA production and enhanced the malignancy of non-small cell lung cancer (NSCLC) cells. Clinically, high expression of DLAT was positively associated with tumor size, poorer prognosis, and SUVmax values of 18F-FDG-PET/CT scans in patients with NSCLC (Chen et al., 2022). GLS the first enzyme in glutaminolysis, is overexpressed in ibrutinib-resistant mantle cell lymphoma (MCL) cells, and that its expression correlates well with elevated glutamine dependency and glutaminolysis. Targeting glutaminase is therapeutically effective in ibrutinib-resistant MCL (Li et al., 2022). PDHA1, a subunit of the pyruvate dehydrogenase complex (PDC), inhibits prostate cancer development in mouse and human xenograft tumor models by affecting lipid biosynthesis (Chen et al., 2018). Therefore, upregulated CRGs has an important biological significance in a variety of human tumors.

Univariate Cox regression and LASSO Cox regression analysis were subsequently used to analyze the five signature genes. The prognostic value of these five genes was then tested, and the results showed that HCC patients with high expression of these five CRGs had decreased survival rates. Moreover, the higher expression of *GLS, DLAT,* and *CDKN2A* increased the risk score, and the OS rate of patients in the high-risk group was significantly shorter than that of the patients in the low-risk group. In addition, *CDKN2A* was found as an independent risk factor, and a valid nomogram was constructed to predict the 1-, 3-, and 5-year survival rates of HCC patients, suggesting that *CDKN2A* played an important role in the occurrence and prognosis of HCC.

Most malignancies are caused by somatic mutations within the cancer genome, and mutational signatures correlate with mRNA and protein expression in multiple cancer types (Weir et al., 2004). Functional alterations due to somatic mutations in cancer genomes are critical for identifying driver mutations and developing molecularly targeted therapies (Jia and Zhao, 2017). In this study, the analysis of the cBioPortal database showed that prognostic CRGs had a high frequency of gene alterations in HCC patients, and the survival rate of HCC patients with mutations in prognostic CRGs was significantly reduced compared with those without mutations. In other words, the mutation of prognostic CRGs in HCC accelerated the progression of HCC, providing some directions for the development of targeted drugs in the treatment of HCC in the future.

The interaction of the tumor and the immune system also plays an important role in the occurrence, development, and treatment of cancer (Kong et al., 2020; Xiao et al., 2020). Immune cells are an important part of the TME, and innate immune cells (including macrophages, neutrophils, dendritic cells, and natural killer cells) and adaptive immune cells (T cells and B cells) play an important role in tumor progression (Thakkar et al., 2020). High CD10^+^/low CD20^+^ immune cell infiltration ratio is an important prognostic factor for lung squamous cell carcinoma (Kadota et al., 2015). Immunomodulatory therapy of tumor-specific neutrophil and B lymphocyte responses may be suitable in the treatment of lung squamous cell carcinoma (Kadota et al., 2015) (65). Correale P’ team investigated the prognostic value of tumor-infiltrating CD8^+^ T cells expressing the chemokine receptor 7 [T(ccr7)], demonstrating that patients with colorectal cancer with high T (ccr7) and T (reg) invasion have a better prognosis (Correale et al., 2012). In the present study, the expression of prognostic CRGs in HCC was significantly and positively correlated with the infiltration of immune cells. Therefore, the target of prognostic CRGs with the aim of interfering with the function of immune cell infiltration in HCC might provide a new solution for immunotherapy against HCC.

At present, immunotherapy targeting immune checkpoints, especially programmed cell death protein 1/programmed cell death ligand 1 (PD-1/PD-L1) and CTLA-4 blockers have become feasible in the treatment of many malignant tumors (Cai et al., 2019). PD-1 is a member of the CD28 family. PD-1 and its ligands are widely expressed in T cells and play a broader immunoregulatory role in T cell activation and tolerance (Cha et al., 2019). PD-L1 is a transmembrane protein considered as a co-suppressor of the immune response, and it binds to PD-1 to reduce the proliferation of PD-1-positive cells, inhibits their cytokine secretion and induce apoptosis. PD-L1 also plays an important role in various malignancies, attenuating the host immune response to tumor cells (Han et al., 2020). The PD-1/PD-L1 axis is responsible for cancer immune escape and has a huge impact on cancer therapy, and its inhibition is an effective treatment for many cancers (Dermani et al., 2019). Previous studies found that copper regulates a key signaling pathway that mediates PD-L1-driven cancer immune evasion (Voli et al., 2020). Cytotoxic T lymphocyte-associated antigen 4 (CTLA-4) is a membrane glycoprotein expressed by activated effector T cells and is involved in the inhibition of T cell proliferation, cell cycle progression and cytokine production (Zhao et al., 2018). Antibodies targeting CTLA-4 or in combination with other therapies significantly enhance the antitumor effects and improve the prognosis in malignant diseases (Zhao et al., 2018). This study found that the expression of prognostic CRGs in the TCGA-LIHC cohort was positively correlated with the expression of PD-1 (PDCD1), PD-L1 (CD-274) and CTLA-4, suggesting that the prognosis of HCC patients with high expression of CRGs should be improved by immunotherapy targeting immune checkpoints.

The research on tumor immune checkpoint inhibitors in tumor immunotherapy is the most developed. TMB and MSI are considered potential predictive biomarkers involved in response to ICIs (Dudley et al., 2016; Ritterhouse, 2019). High TMB is associated with the response to ICI in multiple tumor types (Weir et al., 2004). Our results showed that *CDKN2A* increased the TMB score in HCC and *PDHA1* increased the MSI score. Furthermore, prognostic CRGs were positively or negatively associated with multiple chemotherapeutic agents. These results provide new potential therapeutic targets in the treatment of HCC.

N6-methyladenosine (m6A) is the most prevalent internal mRNA modification in mammalian cells. RNA methylation is a pervasive post-transcriptional modification that plays a key role in regulating various biological processes, and its dysregulation is closely related to the occurrence of human malignancies (Anita et al., 2020; Chen and Wong, 2020; Han et al., 2021; Liu et al., 2021; Zhang et al., 2021) through various mechanisms, providing more possibilities for the early diagnosis and treatment of cancer (Sun et al., 2019). METTL3 is involved in pancreatic carcinogenesis and is a potential prognostic marker or therapeutic target (Xia et al., 2019). HBXIP promotes the progression of gastric cancer *via* METTL3-mediated MYC mRNA m6A modification (Yang et al., 2020). Currently, the link between CRGs and m6A-related genes in HCC has not been investigated, which was one of the aims of the present study. Our result showed that m6A-related genes were significantly upregulated in the C1 cluster with higher expression of CRGs, and prognostic CRGs were significantly correlated with m6A-related genes, suggesting that CRGs might affect the progression of HCC through m6A modification. However, further studies should be performed to confirm this result.

Stromal components, including CAFs and TAMs, play important roles in cancer initiation and progression (Hanahan and Coussens, 2012). Our results showed that prognostic CRGs upregulated the expression of macrophages, fibroblasts, endothelial cells, and hepatic progenitor cells in HCC. Furthermore, prognostic CRGs were positively correlated with many markers of CAFs and macrophages. Previous studies showed that CAFs promote the progression of HCC (Ju et al., 2009; Affo et al., 2021). Clinical studies and experimental mouse models also strongly suggest that TAMs promote tumor progression (Qian and Pollard, 2010; Noy and Pollard, 2014). The present study found that prognostic CRGs upregulated the expression of macrophages, fibroblasts, endothelial cells, and hepatic progenitor cells in HCC. Moreover, prognostic CRGs were significantly and positively correlated with many markers of stromal cells, especially CAFs and TAMs. Therefore, prognostic CRGs might affect the progression of HCC patients by altering the expression of CAFs and TAMs in the TME.

Eight lncRNA-miRNA-mRNA networks were also constructed, lncRNA DANCR reduces the expression of miR-125b-5p and regulates the proliferation and apoptosis of hepatoma cells (Yang et al., 2021). LncRNA CYTOR affects the proliferation, cell cycle and apoptosis of hepatoma cells by regulating the miR-125b5p/KIAA1522 axis (Bo et al., 2021). LncRNA DLEU2 aggravates the progression of HCC (Guo et al., 2019). Furthermore, the GSEC/miR-101-3p/SNX16/PAPOLG axis of the ceRNA network axis is an important factor associated with HCC prognosis and immune infiltration (Hu et al., 2022). Our study revealed that these mRNA-miRNA-lncRNA networks were associated with the prognosis of HCC patients. All these pieces of evidence suggest that these regulatory axes might play an important role in the progression of HCC. However, further studies should be performed to confirm this result.

## Conclusion

CRGs affected the infiltration of immune cells such as macrophages and CD8^+^ T cells, promoted the expression of immune checkpoints such as CD274 and HAVCR2, and promoted the expression of various m6A-related genes in HCC. CRGs also participated in multiple cancer-related signaling pathways in HCC. A total of 19 CRGs that were highly expressed in HCC were analyzed, and five CRGs (*CDKN2A, DLAT, DLST, GLS,* and *PDHA1*) that were associated with the prognosis of HCC patients were identified. A prognostic model of CRGs was established, which predicted more accurately OS and PFS in HCC patients. Mutations in prognostic CRGs led to poorer prognosis in patients with HCC. Prognostic CRGs were positively correlated with B cells, T cells, macrophages, and other immune cells, and were positively correlated with various chemotherapeutic drugs. They affected the TME in HCC by affecting CAFs and TAMs. Finally, eight lncRNA-miRNA-mRNA regulatory axes that affected the progression of HCC were predicted. Hence, our study laid the foundation for an in-depth understanding of the role of CRGs in HCC.

## Data Availability

The datasets are available in TCGA database (https://portal.gdc.cancer.gov/), GDSC database (https://www.cancerrxgene.org/), GeneMANIA (http://www.genemania.org), GSEA (http://software.broadinstitute.org/gsea/index.jsp), Human Protein Atlas database (https://www.proteinatlas.org), cBioPortal (http://www.cbioportal.org/), GDSC database (https://www.cancerrxgene.org/), GEPIA2 database (http://gepia2.cancer-pku.cn/#index), TISIDB (http://cis.hku.hk/TISIDB), TISCH database (http://tisch.comp-genomics.org/), ENCORI database (http://starbase.sysu.edu.cn/), RNAInter database (http://www.rnainter.org/), miRNet database (http://www.mirnet.ca/), RNA22 database (https://cm.jefferson.edu/rna22/interactive) as well as TIMER database (https://cistrome.shinyapps.io/timer/), UALCAN database (http://ualcan.path.uab.edu/).

## References

[B1] Affo S. Nair A. Brundu F. Ravichandra A. Bhattacharjee S. Matsuda M. (2021). Promotion of cholangiocarcinoma growth by diverse cancer-associated fibroblast subpopulations. Cancer Cell 39 (6), 866–882.e11. 10.1016/j.ccell.2021.03.012 33930309 PMC8241235

[B2] Ai L. Stephenson K. K. Ling W. Zuo C. Mukunyadzi P. Suen J. Y. (2003). The p16 (CDKN2a/INK4a) tumor-suppressor gene in head and neck squamous cell carcinoma: A promoter methylation and protein expression study in 100 cases. Mod. Pathol. 16 (9), 944–950. 10.1097/01.MP.0000085760.74313 13679459

[B3] Anderson N. M. Qin X. Finan J. M. Lam A. Athoe J. Missiaen R. (2021). Metabolic enzyme DLST promotes tumor aggression and reveals a vulnerability to OXPHOS inhibition in high-risk neuroblastoma. Cancer Res. 81 (17), 4417–4430. 10.1158/0008-5472.Can-20-2153 34233924 PMC8577318

[B4] Anita R. Paramasivam A. Priyadharsini J. V. Chitra S. (2020). The m6A readers YTHDF1 and YTHDF3 aberrations associated with metastasis and predict poor prognosis in breast cancer patients. Am. J. Cancer Res. 10 (8), 2546–2554. 32905518 PMC7471347

[B5] Aran D. Hu Z. Butte A. J. (2017). xCell: digitally portraying the tissue cellular heterogeneity landscape. Genome Biol. 18 (1), 220. 10.1186/s13059-017-1349-1 29141660 PMC5688663

[B6] Baharvand M. Manifar S. Akkafan R. Mortazavi H. Sabour S. (2014). Serum levels of ferritin, copper, and zinc in patients with oral cancer. Biomed. J. 375, 331–336. 10.4103/2319-4170.132888 25179706

[B7] Becht E. Giraldo N. A. Lacroix L. Buttard B. Elarouci N. Petitprez F. (2016). Estimating the population abundance of tissue-infiltrating immune and stromal cell populations using gene expression. Genome Biol. 17 (1), 218. 10.1186/s13059-016-1070-5 27765066 PMC5073889

[B8] Bian Z. Fan R. Xie L. (2022). A novel cuproptosis-related prognostic gene signature and validation of differential expression in clear cell renal cell carcinoma. Genes (Basel) 13 (5), 851. 10.3390/genes13050851 35627236 PMC9141858

[B9] Bo H. XiaoBo Y. Xu Y. XinTing S. (2021). LncRNA CYTOR affects the proliferation, cell cycle and apoptosis of hepatocellular carcinoma cells by regulating the miR-125b-5p/KIAA1522 axis. Aging(Albany NY) 13 (2), 2626–2639. 10.18632/aging.202306 PMC7880333 33318318

[B10] Brady D. C. Crowe M. S. Turski M. L. Hobbs G. A. Yao X. Chaikuad A. (2014). Copper is required for oncogenic BRAF signalling and tumorigenesis. Nature 5097501, 492–496. 10.1038/nature13180 PMC4138975 24717435

[B11] Cai J. Wang D. Zhang G. Guo X. (2019). The role of PD-1/PD-L1 Axis in treg development and function: Implications for cancer immunotherapy. Onco Targets Ther. 12, 8437–8445. 10.2147/OTT.S221340 31686860 PMC6800566

[B12] Cha J. H. Chan L. C. Li C. W. Hsu J. L. Hung M. C. (2019). Mechanisms controlling PD-L1 expression in cancer. Mol. Cell 76 (3), 359–370. 10.1016/j.molcel.2019.09.030 31668929 PMC6981282

[B13] Chang L. Zhou G. Soufan O. Xia J. (2020). miRNet 2.0: network-based visual analytics for miRNA functional analysis and systems biology. Nucleic Acids Res. 48, W244–W251. 10.1093/nar/gkaa467 32484539 PMC7319552

[B14] Chen J. Guccini I. Di Mitri D. Brina D. Revandkar A. Sarti M. (2018). Publisher Correction: Compartmentalized activities of the pyruvate dehydrogenase complex sustain lipogenesis in prostate cancer. Nat. Genet. 50 (9), 1343. 10.1038/s41588-018-0181-1 30089860

[B15] Chen M. Wong C. M. (2020). The emerging roles of N6-methyladenosine (m6A) deregulation in liver carcinogenesis. Mol. Cancer 19 (1), 44. 10.1186/s12943-020-01172-y 32111216 PMC7047367

[B16] Chen Q. Wang Y. Yang L. Sun L. Wen Y. Huang Y. (2022). PM2.5 promotes NSCLC carcinogenesis through translationally and transcriptionally activating DLAT-mediated glycolysis reprograming. J. Exp. Clin. cancer Res. CR 41 (1), 229. 10.1186/s13046-022-02437-8 35869499 PMC9308224

[B17] Christodoulou E. Nell Q. J. Verdijk R. M. Gruis N. A. Velden P. A. Doorn R. V. (2020). Loss of wild-type CDKN2A is an early event in the development of melanoma in FAMMM syndrome. J. Invest. Dermatol 14011, 2298–2301.e3. 10.1016/j.jid.2020.03.938 32234459

[B18] Correale P. Rotundo M. S. Botta C. Del Vecchio M. T. Ginanneschi C. Licchetta A. (2012). Tumor infiltration by T lymphocytes expressing chemokine receptor 7 (CCR7) is predictive of favorable outcome in patients with advanced colorectal carcinoma. Clin. Cancer Res. 18 (3), 850–857. 10.1158/1078-0432.CCR-10-3186 22142823

[B19] Czlonkowska A. Litwin T. Dusek P. Ferenci P. Lutsenko S. Medici V. (2018). Wilson disease. Nat. Rev. Dis. Prim. 4 (1), 21. 10.1038/s41572-018-0018-3 30190489 PMC6416051

[B20] Davis C. I. Gu X. Kiefer R. M. Ralle M. Gade T. P. Brady D. C. (2020). Altered copper homeostasis underlies sensitivity of hepatocellular carcinoma to copper chelation. Metallomics 1212, 1995–2008. 10.1039/d0mt00156b PMC8315290 33146201

[B21] Dermani F. K. Samadi P. Rahmani G. Kohlan A. K. Najafi R. (2019). PD-1/PD-L1 immune checkpoint: Potential target for cancer therapy. J. Cell Physiol. 234 (2), 1313–1325. 10.1002/jcp.27172 30191996

[B22] Dragutinović V. V. Tatić S. B. Nikolić-Mandić S. D. Tripković T. M. Dunđerović D. M. Paunović I. R. (2014). Copper as ancillary diagnostic tool in preoperative evaluation of possible papillary thyroid carcinoma in patients with benign thyroid disease. Biol. Trace Elem. Res. 1603, 311–315. 10.1007/s12011-014-0071-z 25035190

[B23] Dudley J. C. Lin M. T. Le D. T. Eshleman J. R. (2016). Microsatellite instability as a biomarker for PD-1 blockade. Clin. Cancer Res. 22 (4), 813–820. 10.1158/1078-0432.CCR-15-1678 26880610

[B24] Eke I. Cordes N. (2015). Focal adhesion signaling and therapy resistance in cancer. Semin. Cancer Biol. 31, 65–75. 10.1016/j.semcancer.2014.07.009 25117005

[B25] Fang Y. Tian S. Pan Y. Li W. Wang Q. Tang Y. (2020). Pyroptosis: A new frontier in cancer. Biomed. Pharmacother. 121, 109595. 10.1016/j.biopha.2019.109595 31710896

[B26] Fendt S. M. Bell E. L. Keibler M. A. Olenchock B. A. Mayers J. R. Wasylenko T. M. (2013). Reductive glutamine metabolism is a function of the alpha-ketoglutarate to citrate ratio in cells. Nat. Commun. 4, 2236. 10.1038/ncomms3236 23900562 PMC3934748

[B27] Finotello F. Mayer C. Plattner C. Laschober G. Rieder D. Hackl H. (2019). Molecular and pharmacological modulators of the tumor immune contexture revealed by deconvolution of RNA-seq data. Genome Med. 11 (1), 34. 10.1186/s13073-019-0638-6 31126321 PMC6534875

[B28] Gao J. J. Aksoy B. A. Dogrusoz U. Dresdner G. Gross B. Sumer S. O. (2013). Integrative analysis of complex cancer genomics and clinical profiles using the cBioPortal. Sci. Signal. 6 (269), pl1. 10.1126/scisignal.2004088 23550210 PMC4160307

[B29] Golonka R. M. Vijay-Kumar M. (2021). Atypical immunometabolism and metabolic reprogramming in liver cancer: Deciphering the role of gut microbiome. Adv. Cancer Res. 149, 171–255. 10.1016/bs.acr.2020.10.004 33579424

[B30] Guo Y. Bai M. Lin L. Huang J. An Y. Liang L. (2019). LncRNA DLEU2 aggravates the progression of hepatocellular carcinoma through binding to EZH2. Biomed. Pharmacother. 118, 109272. 10.1016/j.biopha.2019.109272 31376657

[B31] Han X. Wang M. Zhao Y. L. Yang Y. Yang Y. G. (2021). RNA methylations in human cancers. Semin. Cancer Biol. 75, 97–115. 10.1016/j.semcancer.2020.11.007 33220459

[B32] Han Y. Y. Liu D. D. Li L. H. (2020). PD-1/PD-L1 pathway: Current researches in cancer. Am. J. Cancer Res. 10 (3), 727–742. 32266087 PMC7136921

[B33] Hanahan D. Coussens L. M. (2012). Accessories to the crime: Functions of cells recruited to the tumor microenvironment. Cancer Cell 21 (3), 309–322. 10.1016/j.ccr.2012.02.022 22439926

[B34] Hanzelmann S. Castelo R. Guinney J. (2013). Gsva: Gene set variation analysis for microarray and RNA-seq data. BMC Bioinforma. 14, 7. 10.1186/1471-2105-14-7 PMC3618321 23323831

[B35] Hellyer J. A. Padda S. K. Diehn M. Wakelee H. A. (2021). Clinical implications of KEAP1-nfe2l2 mutations in NSCLC. J. Thorac. Oncol. 16 (3), 395–403. 10.1016/j.jtho.2020.11.015 33307193

[B36] Hu S. Zhang J. Guo G. Zhang L. Dai J. Gao Y. (2022). Comprehensive analysis of GSEC/miR-101-3p/SNX16/PAPOLG axis in hepatocellular carcinoma. PLoS One 17 (4), e0267117. 10.1371/journal.pone.0267117 35482720 PMC9049542

[B37] Jemal A. Ward E. M. Johnson C. J. Cronin K. A. Ma J. M. Ryerson A. B. (2017). Annual report to the nation on the status of cancer, 1975-2014, featuring survival. J. Natl. Cancer Inst. 1099, djx030. 10.1093/jnci/djx030 PMC5409140 28376154

[B38] Ji Z. Huo C. Yang P. (2020). Genistein inhibited the proliferation of kidney cancer cells via CDKN2a hypomethylation: Role of abnormal apoptosis. Int. Urol. Nephrol. 52 (6), 1049–1055. 10.1007/s11255-019-02372-2 32026308

[B39] Jia P. Zhao Z. (2017). Impacts of somatic mutations on gene expression: An association perspective. Brief. Bioinform 18 (3), 413–425. 10.1093/bib/bbw037 27127206 PMC5862283

[B40] Ju M. Bi J. Wei Q. Jiang L. Guan Q. Zhang M. (2021). Pan-cancer analysis of NLRP3 inflammasome with potential implications in prognosis and immunotherapy in human cancer. Brief. Bioinform 22 (4), bbaa345. 10.1093/bib/bbaa345 33212483 PMC8294515

[B41] Ju M. J. Qiu S. J. Fan J. Xiao Y. S. Gao Q. Zhou J. (2009). Peritumoral activated hepatic stellate cells predict poor clinical outcome in hepatocellular carcinoma after curative resection. Am. J. Clin. Pathol. 131 (4), 498–510. 10.1309/AJCP86PPBNGOHNNL 19289585

[B42] Kaba M. Pirincci N. Yuksel M. B. Gecit I. Gunes M. Demir M. (2015). Serum levels of trace elements in patients with testicular cancers. Int. Braz J. Urol. 416, 1101–1107. 10.1590/S1677-5538.IBJU.2014.0460 PMC4756935 26742967

[B43] Kadota K. Nitadori J. I. Ujiie H. Buitrago D. H. Woo K. M. Sima C. S. (2015). Prognostic impact of immune microenvironment in lung squamous cell carcinoma: Tumor-infiltrating CD10+ neutrophil/cd20+ lymphocyte ratio as an independent prognostic factor. J. Thorac. Oncol. 10 (9), 1301–1310. 10.1097/JTO.0000000000000617 26291010 PMC4545576

[B44] Kong X. Fu M. Niu X. Jiang H. (2020). Comprehensive analysis of the expression, relationship to immune infiltration and prognosis of TIM-1 in cancer. Front. Oncol. 10, 1086. 10.3389/fonc.2020.01086 33014768 PMC7498659

[B45] Le A. Lane A. N. Hamaker M. Bose S. Gouw A. Barbi J. (2012). Glucose-independent glutamine metabolism via TCA cycling for proliferation and survival in B cells. Cell Metab. 151, 110–121. 10.1016/j.cmet.2011.12.009 PMC3345194 22225880

[B46] Li B. Severson E. Pignon J. C. Zhao H. Li T. Novak J. (2016). Comprehensive analyses of tumor immunity: Implications for cancer immunotherapy. Genome Biol. 17 (1), 174. 10.1186/s13059-016-1028-7 27549193 PMC4993001

[B47] Li D. Li Y. (2020). The interaction between ferroptosis and lipid metabolism in cancer. Signal Transduct. Target Ther. 51, 108. 10.1038/s41392-020-00216-5 PMC7327075 32606298

[B48] Li H. Gao Y. Ren C. (2021). Focal adhesion kinase inhibitor BI 853520 inhibits cell proliferation, migration and EMT process through PI3K/AKT/mTOR signaling pathway in ovarian cancer. Discov. Oncol. 12 (1), 29. 10.1007/s12672-021-00425-6 35201437 PMC8777525

[B49] Li J. H. Liu S. Zhou H. Qu L. H. Yang J. H. (2014). starBase v2.0: decoding miRNA-ceRNA, miRNA-ncRNA and protein-RNA interaction networks from large-scale CLIP-Seq data. Nucleic Acids Res. 42, D92–D97. 10.1093/nar/gkt1248 24297251 PMC3964941

[B50] Li L. Nie L. Jordan A. Cai Q. Liu Y. Li Y. (2022). Targeting glutaminase is therapeutically effective in ibrutinib-resistant mantle cell lymphoma. Haematologica. Advance online publication. 10.3324/haematol.2022.281538 PMC10230437 36420799

[B51] Li T. Fan J. Wang B. Traugh N. Chen Q. Liu J. S. (2017). Timer: A web server for comprehensive analysis of tumor-infiltrating immune cells. Cancer Res. 77 (21), e108–e110. 10.1158/0008-5472.CAN-17-0307 29092952 PMC6042652

[B52] Lin Y. Liu T. Cui T. Wang Z. Zhang Y. Tan P. (2020). RNAInter in 2020: RNA interactome repository with increased coverage and annotation. Nucleic Acids Res. 48 (D1), D189–D197. 10.1093/nar/gkz804 31906603 PMC6943043

[B53] Liu Z. He J. Han J. Yang J. Liao W. Chen N. (2021). m6A regulators mediated methylation modification patterns and tumor microenvironment infiltration characterization in nasopharyngeal carcinoma. Front. Immunol. 12, 762243. 10.3389/fimmu.2021.762243 35069534 PMC8776994

[B54] Liver E. A. Cancer E. O. (2012). EASL-EORTC clinical practice guidelines: Management of hepatocellular carcinoma. J. Hepatol. 564, 908–943. 10.1016/j.jhep.2011.12.001 22424438

[B55] Llovet J. M. Zucman-Rossi J. Pikarsky E. Sangro B. Schwartz M. Sherman M. (2016). Hepatocellular carcinoma. Nat. Rev. Dis. Prim. 71, 16018. 10.1038/nrdp.2016.18 27158749

[B56] Loher P. Rigoutsos I. (2012). Interactive exploration of RNA22 microRNA target predictions. Bioinformatics 28 (24), 3322–3323. 10.1093/bioinformatics/bts615 23074262

[B57] Lutsenko S. (2010). Human copper homeostasis: A network of interconnected pathways. Curr. Opin. Chem. Biol. 142, 211–217. 10.1016/j.cbpa.2010.01.003 PMC6365103 20117961

[B58] Militello G. Weirick T. John D. Döring C. Dimmeler S. Uchida S. (2017). Screening and validation of lncRNAs and circRNAs as miRNA sponges. Brief. Bioinform 18 (5), 780–788. 10.1093/bib/bbw053 27373735

[B59] Nevitt T. Ohrvik H. Thiele D. J. (2012). Charting the travels of copper in eukaryotes from yeast to mammals. Biochim. Biophys. Acta 1823 (9), 1580–1593. 10.1016/j.bbamcr.2012.02.011 22387373 PMC3392525

[B60] Newman A. M. Liu C. L. Green M. R. Gentles A. J. Feng W. Xu Y. (2015). Robust enumeration of cell subsets from tissue expression profiles. Nat. methods 12 (5), 453–457. 10.1038/nmeth.3337 25822800 PMC4739640

[B61] Noy R. Pollard J. W. (2014). Tumor-associated macrophages: From mechanisms to therapy. Immunity 41 (1), 49–61. 10.1016/j.immuni.2014.06.010 25035953 PMC4137410

[B62] Ozden O. Park S. H. Wagner B. A. Song H. Y. Zhu Y. Vassilopoulos A. (2014). SIRT3 deacetylates and increases pyruvate dehydrogenase activity in cancer cells. Free Radic. Biol. Med. 76, 163–172. 10.1016/j.freeradbiomed.2014.08.001 25152236 PMC4364304

[B63] Powers R. K. Goodspeed A. Pielke-Lombardo H. Tan A. C. Costello J. C. (2018). GSEA-InContext: Identifying novel and common patterns in expression experiments. Bioinformatics 34 (13), i555–i564. 10.1093/bioinformatics/bty271 29950010 PMC6022535

[B64] Qian B. Z. Pollard J. W. (2010). Macrophage diversity enhances tumor progression and metastasis. Cell 141 (1), 39–51. 10.1016/j.cell.2010.03.014 20371344 PMC4994190

[B65] Racle J. de Jonge K. Baumgaertner P. Speiser D. E. Gfeller D. (2017). Simultaneous enumeration of cancer and immune cell types from bulk tumor gene expression data. eLife 6, e26476. 10.7554/eLife.26476 29130882 PMC5718706

[B66] Ritchie M. E. Phipson B. Wu D. Hu Y. Law C. W. Shi W. (2015). Limma powers differential expression analyses for RNA-sequencing and microarray studies. Nucleic Acids Res. 43 (7), e47. 10.1093/nar/gkv007 25605792 PMC4402510

[B67] Ritterhouse L. L. (2019). Tumor mutational burden. Cancer Cytopathol. 127 (12), 735–736. 10.1002/cncy.22174 31433548

[B68] Rizzo A. Ricci A. D. Brandi G. (2021). PD-L1, TMB, MSI, and other predictors of response to immune checkpoint inhibitors in biliary tract cancer. Cancers (Basel) 13 (3), 558. 10.3390/cancers13030558 33535621 PMC7867133

[B69] Roth G. S. Decaens T. (2017). Liver immunotolerance and hepatocellular carcinoma: Patho-physiological mechanisms and therapeutic perspectives. Eur. J. Cancer 87, 101–112. 10.1016/j.ejca.2017.10.010 29145036

[B70] Ru B. Wong C. N. Tong Y. Zhong J. Y. Zhong S. Wu W. C. (2019). Tisidb: An integrated repository portal for tumor-immune system interactions. Bioinformatics 35 (20), 4200–4202. 10.1093/bioinformatics/btz210 30903160

[B71] Safi R. Nelson E. R. Chitneni S. K. Franz K. J. George D. J. Zalutsky M. R. (2014). Copper signaling axis as a target for prostate cancer therapeutics. Cancer Res. 7420, 5819–5831. 10.1158/0008-5472.CAN-13-3527 PMC4203427 25320179

[B72] Seong J. Wang N. Wang Y. (2013). Mechanotransduction at focal adhesions: From physiology to cancer development. J. Cell Mol. Med. 17 (5), 597–604. 10.1111/jcmm.12045 23601032 PMC3665742

[B73] Serrano M. (1997). The tumor suppressor protein p16INK4a. Exp. Cell Res. 237, 7–13. 10.1006/excr.1997.3824 9417860

[B74] Shannon P. Markiel A. Ozier O. Baliga N. S. Wang J. T. Ramage D. (2003). Cytoscape: A software environment for integrated models of biomolecular interaction networks. Genome Res. 13 (11), 2498–2504. 10.1101/gr.1239303 14597658 PMC403769

[B75] Song L. Liu D. Zhang X. Zhu X. Lu X. Huang J. (2019). Low expression of PDHA1 predicts poor prognosis in gastric cancer. Pathol. Res. Pract. 215, 478–482. 10.1016/j.prp.2018.12.038 30611622

[B76] Sturm G. Finotello F. List M. (2020). Immunedeconv: An R package for unified access to computational methods for estimating immune cell fractions from bulk RNA-sequencing data. Methods Mol. Biol. 2120, 223–232. 10.1007/978-1-0716-0327-7_16 32124323

[B77] Sun D. Wang J. Han Y. Dong X. Ge J. Zheng R. (2021). Tisch: A comprehensive web resource enabling interactive single-cell transcriptome visualization of tumor microenvironment. Nucleic Acids Res. 49, D1420–D1430. 10.1093/nar/gkaa1020 33179754 PMC7778907

[B78] Sun T. Wu R. Ming L. (2019). The role of m6A RNA methylation in cancer. Biomed. Pharmacother. 112, 108613. 10.1016/j.biopha.2019.108613 30784918

[B79] Tan Y. F. Wang M. Chen Z. Y. Wang L. Liu X. H. (2020). Inhibition of BRD4 prevents proliferation and epithelial-mesenchymal transition in renal cell carcinoma via NLRP3 inflammasome-induced pyroptosis. Cell Death Dis. 114, 239. 10.1038/s41419-020-2431-2 PMC7165180 32303673

[B80] Tang Z. Li C. Kang B. Gao G. Li C. Zhang Z. (2017). Gepia: A web server for cancer and normal gene expression profiling and interactive analyses. Nucleic Acids Res. 45, W98–W102. 10.1093/nar/gkx247 28407145 PMC5570223

[B81] Thakkar S. Sharma D. Kalia K. Tekade R. K. (2020). Tumor microenvironment targeted nanotherapeutics for cancer therapy and diagnosis: A review. Acta Biomater. 101, 43–68. 10.1016/j.actbio.2019.09.009 31518706

[B82] Tomczak K. Czerwińska P. Wiznerowicz M. (2015). The cancer genome Atlas (TCGA): An immeasurable source of knowledge. Contemp. Oncol. Pozn. 19, A68–A77. 10.5114/wo.2014.47136 25691825 PMC4322527

[B83] Tsvetkov P. Coy S. Petrova B. Dreishpoon M. Verma A. Abdusamad M. (2022). Copper induces cell death by targeting lipoylated TCA cycle proteins. Science 375, 1254–1261. 10.1126/science.abf0529 35298263 PMC9273333

[B84] Villanueva A. (2019). Hepatocellular carcinoma. N. Engl. J. Med. 380, 1450–1462. 10.1056/NEJMra1713263 30970190

[B85] Voli F. Valli E. Lerra L. Kimpton K. Saletta F. Giorgi F. M. (2020). Intratumoral copper modulates PD-L1 expression and influences tumor immune evasion. Cancer Res. 80 (19), 4129–4144. 10.1158/0008-5472.CAN-20-0471 32816860

[B86] Warde-Farley D. Donaldson S. L. Comes O. Zuberi K. Badrawi R. Chao P. (2010). The GeneMANIA prediction server: Biological network integration for gene prioritization and predicting gene function. Nucleic Acids Res. 38, W214–W220. 10.1093/nar/gkq537 20576703 PMC2896186

[B87] Weir B. Zhao X. Meyerson M. (2004). Somatic alterations in the human cancer genome. Cancer Cell 6 (5), 433–438. 10.1016/j.ccr.2004.11.004 15542426

[B88] Wilkerson M. D. Hayes D. N. (2010). ConsensusClusterPlus: A class discovery tool with confidence assessments and item tracking. Bioinformatics 26 (12), 1572–1573. 10.1093/bioinformatics/btq170 20427518 PMC2881355

[B89] Xande J. G. Dias A. P. Tamura R. E. Cruz M. C. Brito B. Ferreira R. A. (2020). Bicistronic transfer of CDKN2A and p53 culminates in collaborative killing of human lung cancer cells *in vitro* and *in vivo* . Gene Ther. 27 (1-2), 51–61. 10.1038/s41434-019-0096-1 31439890

[B90] Xia T. Wu X. Cao M. Zhang P. Shi G. Zhang J. (2019). The RNA m6A methyltransferase METTL3 promotes pancreatic cancer cell proliferation and invasion. Pathol. Res. Pract. 215 (11), 152666. 10.1016/j.prp.2019.152666 31606241

[B91] Xiao Z. Hu L. Yang L. Wang S. Gao Y. Zhu Q. (2020). TGFβ2 is a prognostic-related biomarker and correlated with immune infiltrates in gastric cancer. J. Cell Mol. Med. 24 (13), 7151–7162. 10.1111/jcmm.15164 32530106 PMC7339175

[B92] Yaman M. Kaya G. Simsek M. (2007). Comparison of trace element concentrations in cancerous and noncancerous human endometrial and ovary tissues. Int. J. Gynecol. cancer official J. Int. Gynecol. Cancer Soc. 171, 220–228. 10.1111/j.1525-1438.2006.00742.x 17291257

[B93] Yang L. Jiang M. N. Liu Y. Wu C. Q. Liu H. (2021). Crosstalk between lncRNA DANCR and miR-125b-5p in HCC cell progression. Tumori 107 (6), 504–513. 10.1177/0300891620977010 33272103

[B94] Yang W. Soares J. Greninger P. Edelman E. J. Lightfoot H. Forbes S. (2013). Genomics of drug sensitivity in cancer (GDSC): A resource for therapeutic biomarker discovery in cancer cells. Nucleic Acids Res. 41, D955–D961. 10.1093/nar/gks1111 23180760 PMC3531057

[B95] Yang Z. Jiang X. D. Li D. M. Jiang X. F. (2020). HBXIP promotes gastric cancer via METTL3-mediated MYC mRNA m6A modification. Aging (Albany NY) 12 (24), 24967–24982. 10.18632/aging.103767 33048840 PMC7803577

[B96] Yu G. Wang L. Han Y. He Q. (2012). clusterProfiler: an R package for comparing biological themes among gene clusters. OMICS 16 (5), 284–287. 10.1089/omi.2011.0118 22455463 PMC3339379

[B97] Zeng H. Jorapur A. Shain A. H. Lang U. E. Torres R. Zhang Y. (2018). Bi-Allelic loss of CDKN2A initiates melanoma invasion via BRN2 activation. Cancer Cell 34 (1), 56–68.e9. 10.1016/j.ccell.2018.05.014 29990501 PMC6084788

[B98] Zhang J. Lou W. (2020). A key mRNA-miRNA-lncRNA competing endogenous RNA triple sub-network linked to diagnosis and prognosis of hepatocellular carcinoma. Front. Oncol. 10, 340. 10.3389/fonc.2020.00340 32257949 PMC7092636

[B99] Zhang L. Yao Y. X. Zhang S. J. Liu Y. Guo H. Ahmed M. (2019). Metabolic reprogramming toward oxidative phosphorylation identifies a therapeutic target for mantle cell lymphoma. Sci. Transl. Med. 11, eaau1167. 10.1126/scitranslmed.aau1167 31068440

[B100] Zhang L. Zhao D. Wang Y. Zhang W. Zhang J. Fan J. (2020). Focal adhesion kinase (FAK) inhibitor‐defactinib suppresses the malignant progression of human esophageal squamous cell carcinoma (ESCC) cells via effective blockade of PI3K/AKT axis and downstream molecular network. Mol. Carcinog. 60 (2), 113–124. 10.1002/mc.23273 33283357

[B101] Zhang Z. Wang Q. Zhang M. Zhang W. Zhao L. Yang C. (2021). Comprehensive analysis of the transcriptome-wide m6A methylome in colorectal cancer by MeRIP sequencing. Epigenetics 16 (4), 425–435. 10.1080/15592294.2020.1805684 32749190 PMC7993153

[B102] Zhao J. Guan J. (2009). Signal transduction by focal adhesion kinase in cancer. Cancer Metastasis Rev. 28 (1-2), 35–49. 10.1007/s10555-008-9165-4 19169797

[B103] Zhao Y. Yang W. Huang Y. Cui R. Li X. Li B. (2018). Evolving roles for targeting CTLA-4 in cancer immunotherapy. Cell Physiol. Biochem. 47 (2), 721–734. 10.1159/000490025 29794465

[B104] Zhong Y. Huang R. Li X. Xu R. Zhou F. Wang J. (2015). Decreased expression of PDHE1α predicts worse clinical outcome in esophageal squamous cell carcinoma. Anticancer Res. 35, 5533–5538. 26408721

