# Supplementary material for: Comprehensive analysis of cuproptosis-related prognostic gene signature and tumor immune microenvironment in HCC
Source: Front Genet. 2023 Feb 20;14:1094793. doi: 10.3389/fgene.2023.1094793 (PMC9986498; doi:10.3389/fgene.2023.1094793)
Supplement: Supplementary file 1 [file DataSheet1.PDF]

Supplementary Material

1 Supplementary Figures

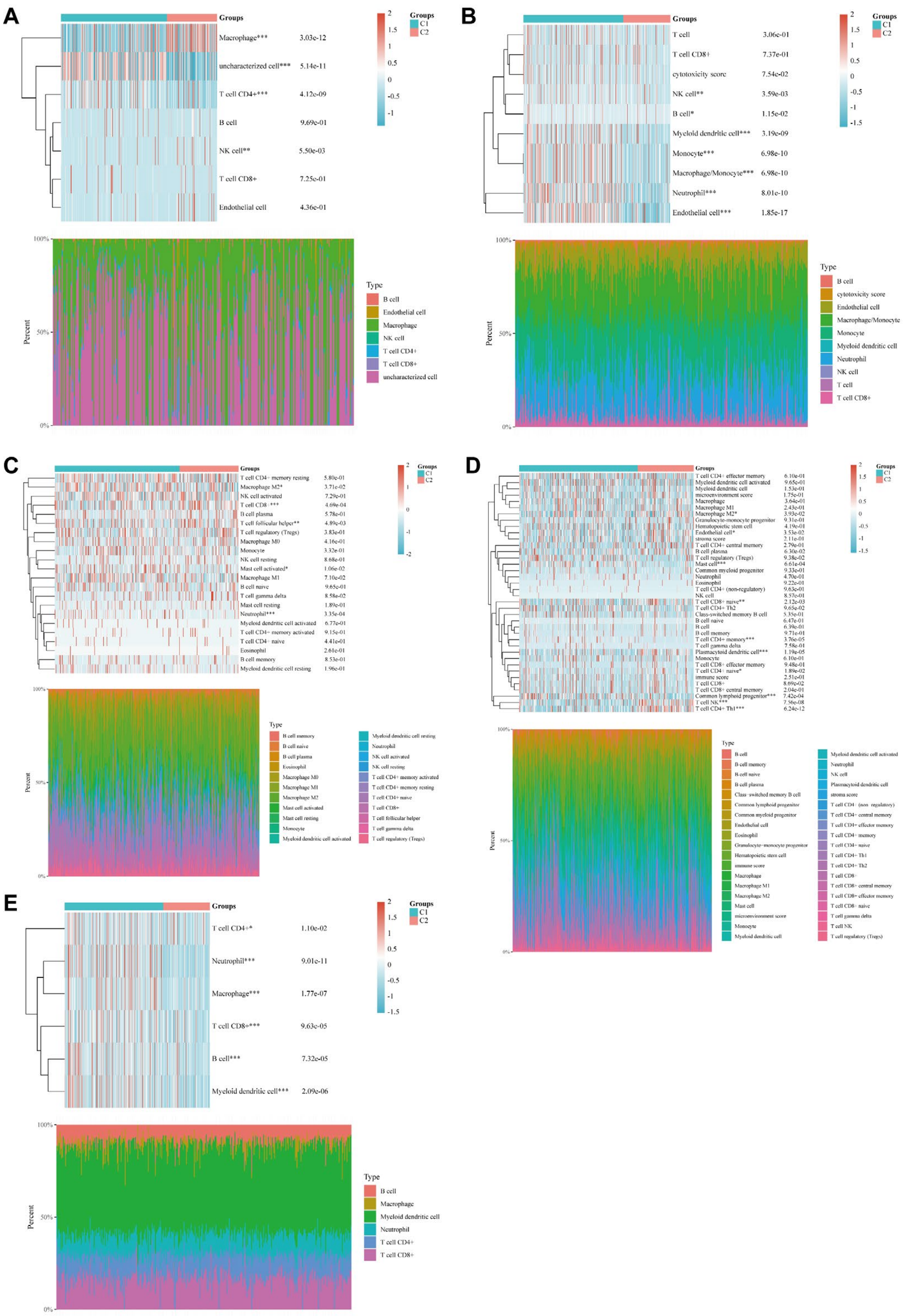

Figure S1: Relationship between CRG expression and immune infiltration in the TME. (A-E) Comparison of immune scores between C1 and C2 subtypes in TCGA (EPIC, MCP-counter, CIBERSORT, xCell and TIMER).

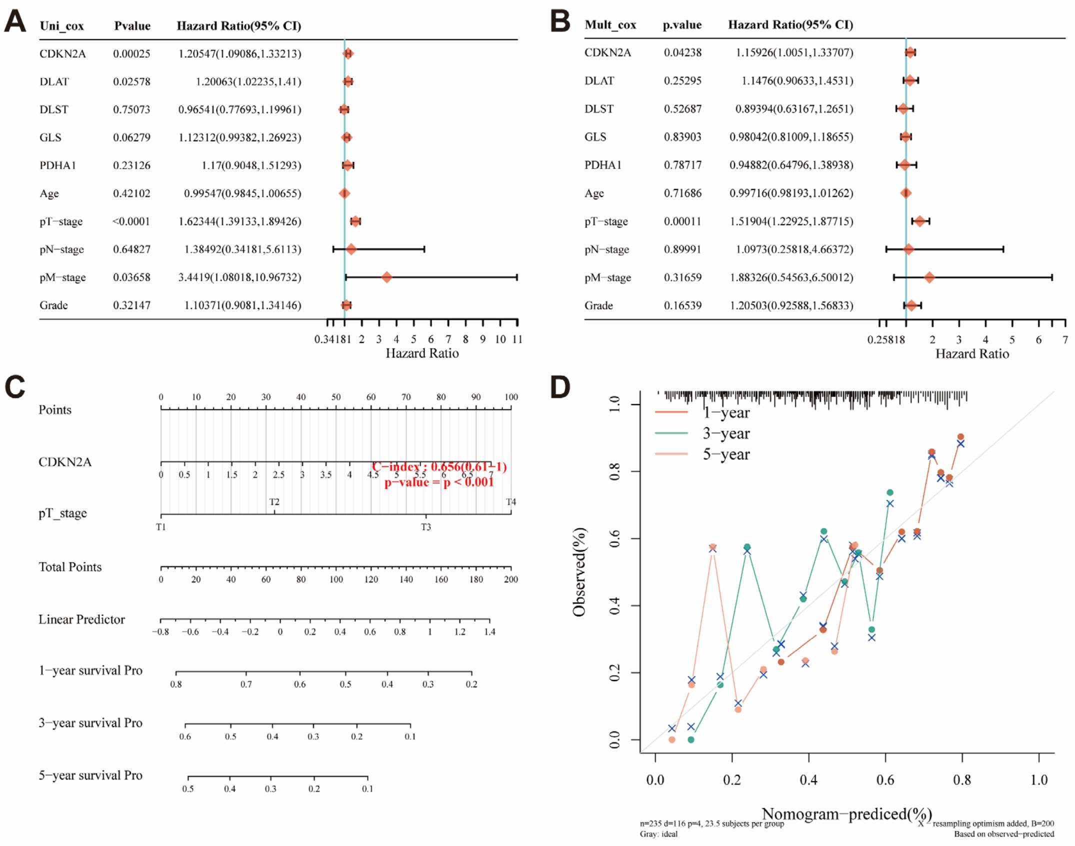

Figure S2: Construction of the predictive nomogram (PFS). (A, B) Hazard ratios and *P* value of the constituents involved in the univariate and multivariate Cox regression analysis considering the clinical information and prognostic CRGs in HCC. (C) Nomogram to predict the 1-year, 3-year, and 5-year PFS rate of HCC patients. (D) Calibration curve for the PFS nomogram model in the discovery group. The dashed diagonal line represents the ideal nomogram. PFS, progression-free survival.

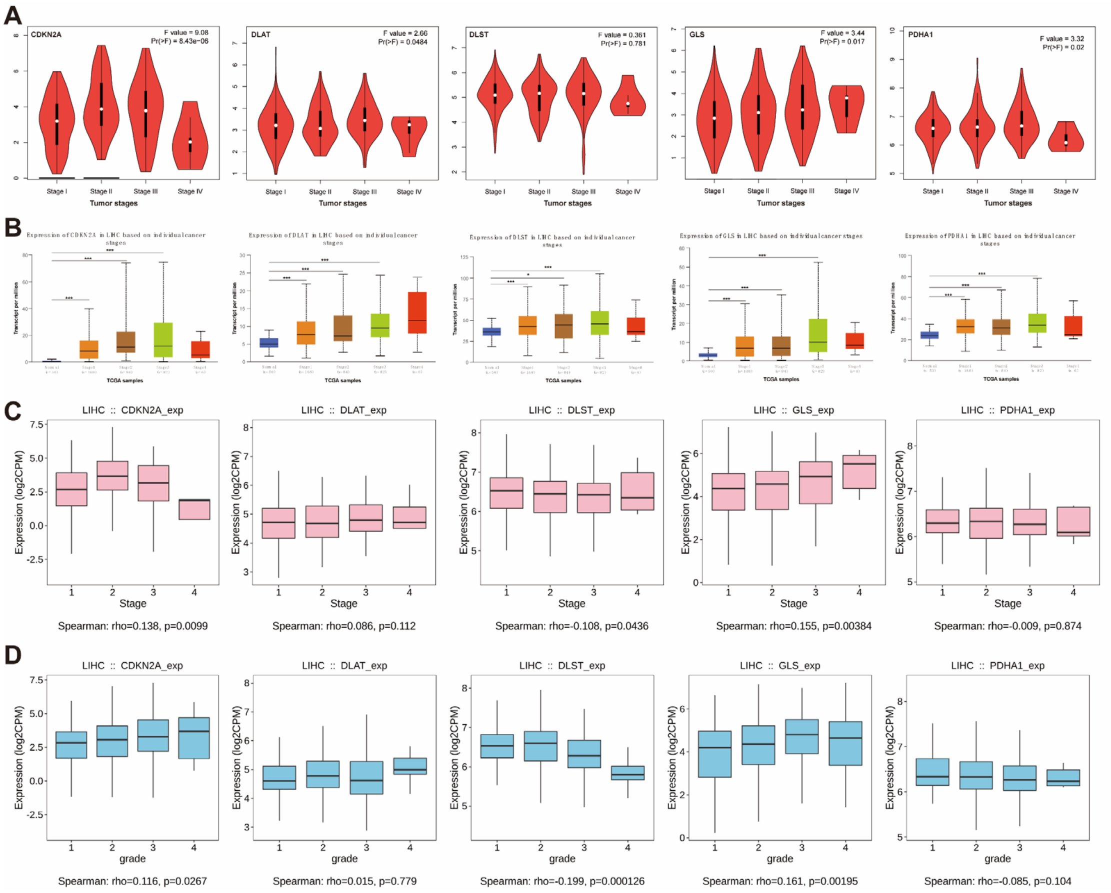

Figure S3: Relationship between prognostic CRGs and pathological stages of HCC analyzed by three databases. (A) Relationship between prognostic CRGs and pathological stage of HCC (I, II, III, IV) in the GEPIA 2 database. (B) Relationship between prognostic CRGs and pathological stage of HCC (I, II, III, IV) in the UALCAN database. (C-D) Relationship between prognostic CRGs and pathological stages and grades (I, II, III, IV) of HCC in the TISIDB database.

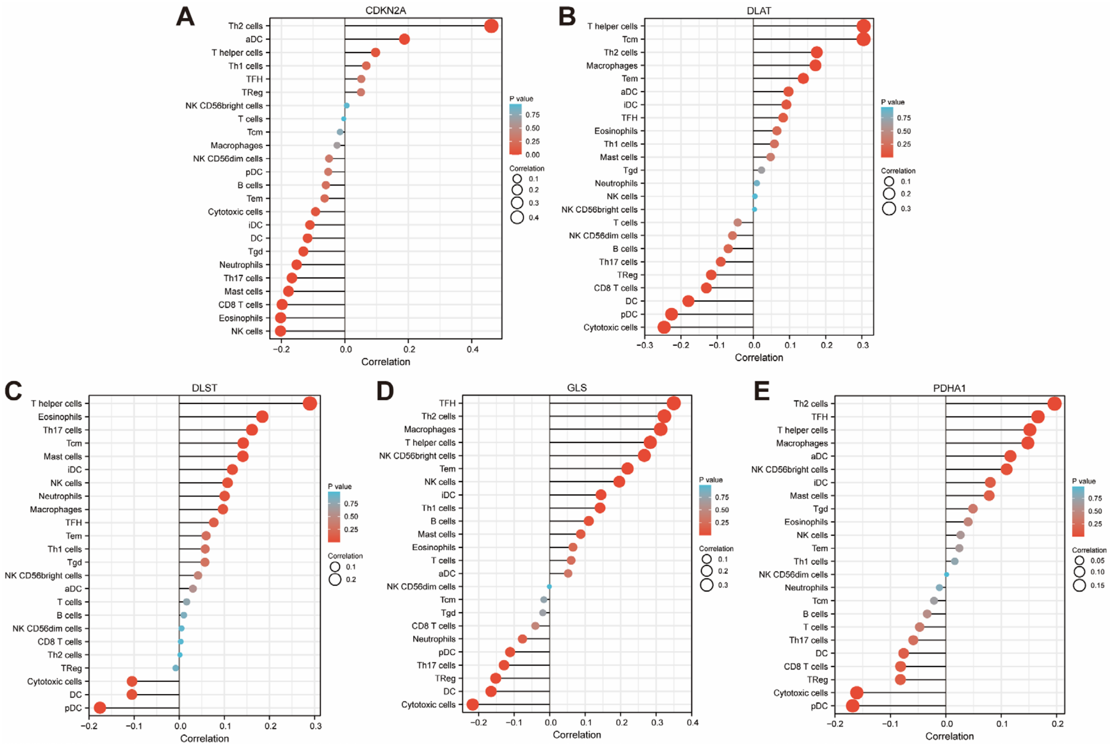

Figure S4: Prognostic CRGs involved in immune infiltration in HCC. (A-E) Association between immune cell abundance and prognostic CRGs in HCC (TCGA database).

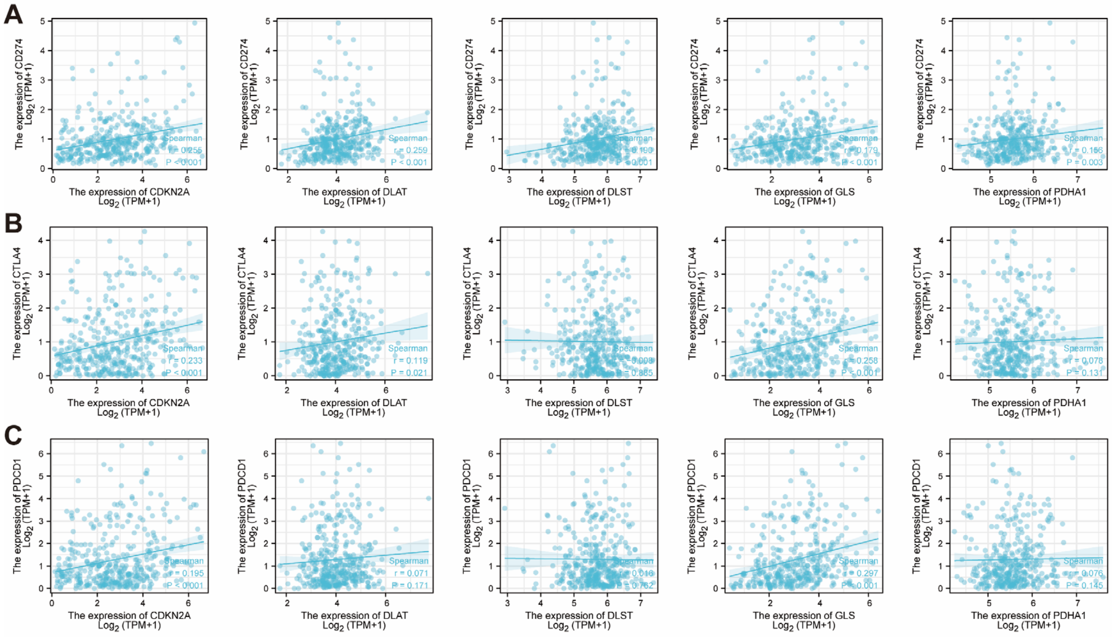

Figure S5: Prognostic CRGs involved in the expression of immune checkpoints in HCC. (A-C) Association between prognostic CRGs and important immune checkpoints (CD274, CTLA4, PDCD1) in HCC.

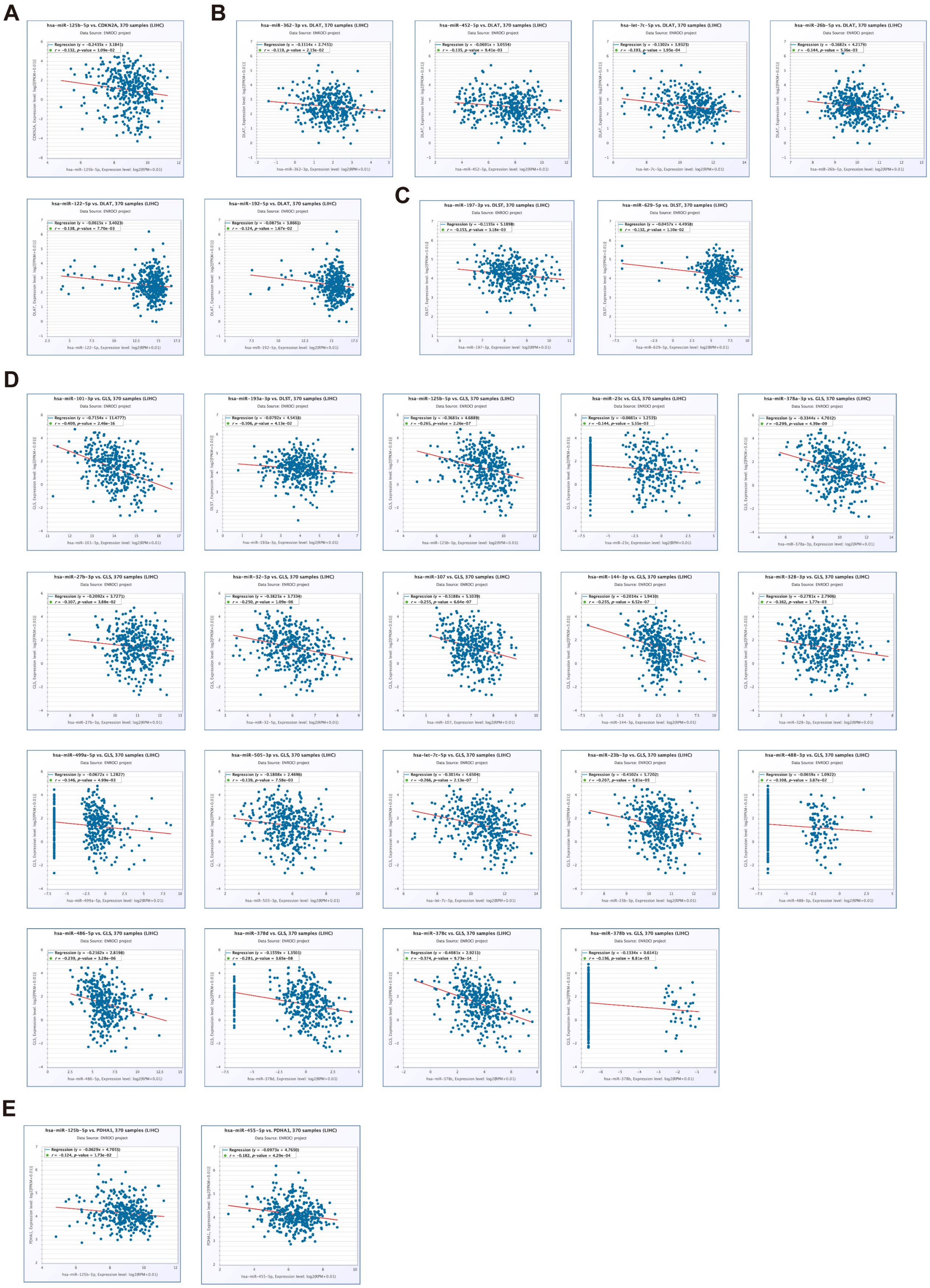

Figure S6: Correlation of prognostic CRGs with potential miRNA targets. (A) CDKN2A vs miRNA. (B) DLAT vs miRNA. (C) DLST vs miRNA. (D) GLS vs miRNA. (E) PDHA1 vs miRNA.

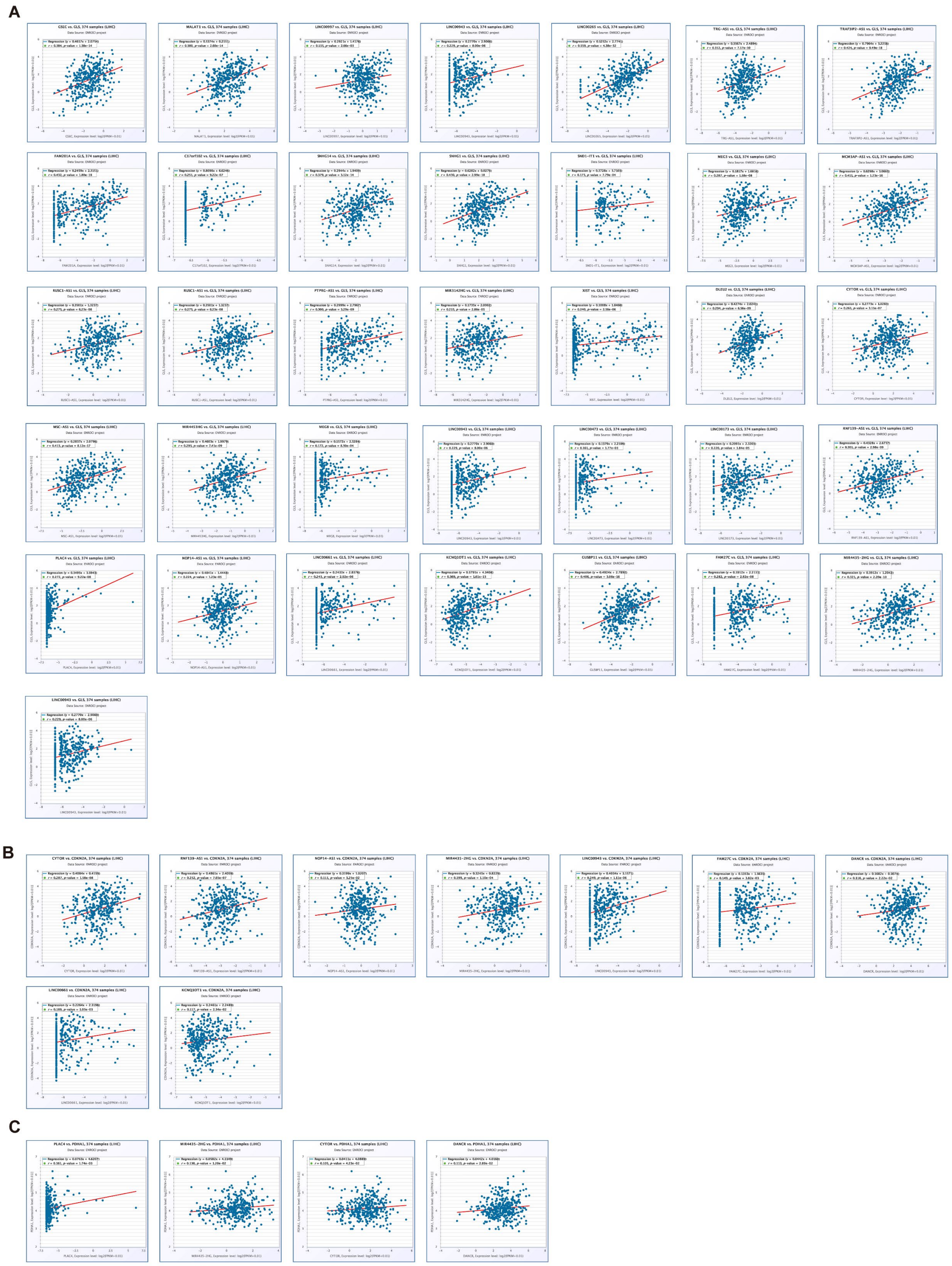

Figure S7: Correlation of prognostic CRGs with potential lncRNA targets. (A) GLS vs lncRNA. (B) CDKN2A vs lncRNA. (C) PDHA1 vs lncRNA.

2 Supplementary Tables

Table S1. Primer sequences of genes

Real-time quantitative PCR primer sequence	
**Gene**	Sequence (5’- 3’ on minus strand)
GAPDH	Fwd: GGAGCGAGATCCCTCCAAAAT
	Rev: GGCTGTTGTCATACTTCTCATGG
CDKN2A	Fwd: ATGGAGCCTTCGGCTGACT
	Rev: GTAACTATTCGGTGCGTTGGG
DLAT	Fwd: CGGAACTCCACGAGTGACC
	Rev: CCCCGCCATACCCTGTAGT
DLST	Fwd: GAACTGCCCTCTAGGGAGAC
	Rev: AACCTTCCTGCTGTTAGGGTA
GLS	Fwd: AGGGTCTGTTACCTAGCTTGG
	Rev: ACGTTCGCAATCCTGTAGATTT
PDHA1	Fwd: TGGTAGCATCCCGTAATTTTGC
	Rev: ATTCGGCGTACAGTCTGCATC

Table S2. lncRNAs significantly associated with hsa-miR-101-3p and GLS.

LncRNA	hsa-miR-101-3p	*GLS*

	R value	p value	R value	p value
RUSC1-AS1	-0.155	2.80×10^{-03}	0.275	6.23×10^{-08}
PTPRG-AS1	-0.189	2.59×10^{-04}	0.300	3.29×10^{-09}
MIR3142HG	-0.265	2.19×10^{-07}	0.215	2.86×10^{-05}
LINC00997	-0.173	8.62×10^{-04}	0.155	2.66×10^{-03}
LINC00265	-0.204	7.71×10^{-05}	0.559	4.38×10^{-32}
SND1-IT1	-0.141	6.44×10^{-03}	0.173	7.79×10^{-04}
FAM201A	-0.259	4.24×10^{-07}	0.432	1.80×10^{-18}
SNHG1	-0.357	1.42×10^{-12}	0.430	2.99×10^{-18}
MALAT1	-0.136	8.76×10^{-03}	0.380	2.60×10^{-14}
GSEC	-0.394	3.37×10^{-15}	0.384	1.38×10^{-14}
LINC00943	-0.137	8.25×10^{-03}	0.229	8.00×10^{-06}
SNHG14	-0.263	2.94×10^{-07}	0.379	3.12×10^{-14}
C17orf102	-0.196	1.54×10^{-04}	0.251	9.22×10^{-07}
RUSC1-AS1	-0.155	2.80×10^{-03}	0.275	6.23×10^{-08}

Table S3. lncRNAs significantly associated with hsa-miR-125b-5p and CDKN2A.

LncRNA	hsa-miR-125b-5p		*CDKN2A*	
	R value	p value	R value	p value
CYTOR	-0.372	1.35×10^{-13}	0.287	1.58×10^{-08}

MIR4435-2HG	-0.307	1.70×10^{-09}	0.199	1.10×10^{-04}
NOP14-AS1	-0.172	9.08×10^{-04}	0.111	3.25×10^{-02}
DANCR	-0.260	3.98×10^{-07}	0.118	2.22×10^{-02}
RNF139-AS1	-0.254	7.32×10^{-07}	0.252	7.65×10^{-07}
FAM27C	-0.181	4.61×10^{-04}	0.149	3.82×10^{-03}
KCNQ1OT1	-0.158	2.36×10^{-03}	0.117	2.34×10^{-02}
LINC00943	-0.137	8.09×10^{-03}	0.249	1.11×10^{-06}
LINC00661	-0.196	1.49×10^{-04}	0.169	1.05×10^{-03}

Table S4. lncRNAs significantly associated with hsa-miR-125b-5p and GLS.

LncRNA	hsa-miR-125b-5p		*GLS*	
	R value	p value	R value	p value
CYTOR	-0.372	1.35×10^{-13}	0.261	3.11×10^{-07}
MIR4435-2HG	-0.307	1.70×10^{-09}	0.321	2.20×10^{-10}
NOP14-AS1	-0.172	9.08×10^{-04}	0.224	1.23×10^{-05}
RNF139-AS1	-0.254	7.32×10^{-07}	0.301	2.98×10^{-09}
FAM27C	-0.181	4.61×10^{-04}	0.282	2.82×10^{-08}
KCNQ1OT1	-0.158	2.36×10^{-03}	0.369	1.61×10^{-13}
LINC00943	-0.137	8.09×10^{-03}	0.229	8.00×10^{-06}
LINC00661	-0.196	1.49×10^{-04}	0.243	2.02×10^{-06}

PLAC4	-0.137	8.42×10^{-03}	0.272	9.22×10^{-08}
GUSBP11	-0.256	6.02×10^{-07}	0.406	3.06×10^{-16}

Table S5. lncRNAs significantly associated with hsa-miR-125b-5p and GLS.

LncRNA	hsa-miR-125b-5p		*PDHA1*	
	R value	p value	R value	p value
CYTOR	-0.372	1.35×10^{-13}	0.105	4.23×10^{-02}
MIR4435-2HG	-0.307	1.70×10^{-09}	0.130	1.20×10^{-02}
DANCR	-0.260	3.98×10^{-07}	0.113	2.89×10^{-02}
PLAC4	-0.137	8.42×10^{-03}	0.161	1.74×10^{-03}

Table S6. lncRNAs were significantly associated with hsa-miR-23c and GLS.

LncRNA	hsa-miR-23c		*GLS*	
	R value	p value	R value	p value
MIR4453HG	-0.114	2.89×10^{-02}	0.293	7.41×10^{-09}
TRAF3IP2-AS1	-0.165	1.48×10^{-03}	0.424	9.49×10^{-18}
LINC00473	-0.195	1.57×10^{-04}	0.161	1.77×10^{-03}
TRG-AS1	-0.134	9.88×10^{-03}	0.312	7.17×10^{-10}
MSC-AS1	-0.152	3.31×10^{-03}	0.413	8.12×10^{-17}
LINC00173	-0.166	1.33×10^{-03}	0.220	1.84×10^{-05}

LINC00943	-0.166	1.38×10^{-03}	0.229	8.00×10^{-06}
DLEU2	-0.117	2.45×10^{-02}	0.294	6.56×10^{-09}
MEG3	-0.114	2.85×10^{-02}	0.287	1.58×10^{-08}
MEG8	-0.122	1.88×10^{-02}	0.172	8.30×10^{-04}
MCM3AP-AS1	-0.119	2.20×10^{-02}	0.411	1.23×10^{-16}
XIST	-0.157	2.41×10^{-03}	0.240	2.56×10^{-06}
